# Germline DNA Retention in Murine and Human Rearranged T Cell Receptor Gene Coding Joints: Alternative Recombination Signal Sequences and V(D)J Recombinase Errors

**DOI:** 10.3389/fimmu.2019.02637

**Published:** 2019-11-08

**Authors:** Justyna Mika, Sylwia Kabacik, Christophe Badie, Joanna Polanska, Serge M. Candéias

**Affiliations:** ^1^Data Mining Division, Silesian University of Technology, Gliwice, Poland; ^2^Cancer Mechanisms and Biomarkers Group, Radiation Effects Department, Centre for Radiation, Chemical and Environmental Hazards Public Health England Chilton, Didcot, United Kingdom; ^3^Université Grenoble Alpes, CEA, CNRS, IRIG-LCBM, Grenoble, France

**Keywords:** T cell receptor genes, V(D)J recombination, RSS, errors, translocations, lymphoid neoplasms

## Abstract

The genes coding for the antigenic T cell receptor (TR) subunits are assembled in thymocytes from discrete V, D, and J genes by a site-specific recombination process. A tight control of this activity is required to prevent potentially detrimental recombination events. V, D, and J genes are flanked by semi-conserved nucleotide motives called recombination signal sequences (RSSs). V(D)J recombination is initiated by the precise introduction of a DNA double-strand break exactly at the border of the genes and their RSSs by the RAG recombinase. RSSs are therefore physically separated from the coding region of the genes before assembly of a rearranged TR gene. During a high throughput profiling of TRB genes in mice, we identified rearranged TRB genes in which part or all of a flanking RSS was retained in V-D or D-J coding joints. In some instances, this retention of germline DNA resulted from the use of an upstream alternative RSS. However, we also identified TRB sequences where retention of germline DNA occurred in the absence of alternative RSS, suggesting that RAG activity was mis-targeted during recombination. Similar events were also identified in human rearranged TRB and TRG genes. The use of alternative RSSs during V(D)J recombination illustrates the complexity of RAG-RSSs interactions during V(D)J recombination. While the frequency of errors resulting from mis-targeted RAG activity is very low, we believe that these RAG errors may be at the origin of oncogenic translocations and are a threat for genetic stability in developing lymphocytes.

## Introduction

T cell receptor (TR) and immunoglobulin (Ig) genes are assembled from discrete V, D, and J gene segments by site-directed rearrangement. This V(D)J recombination process relies on the simultaneous expression of the proteins encoded by the recombination activating gene (RAG)-1 and RAG-2 (1, 2), which form a tetrameric recombinase complex (RAG) constituted of two RAG-1/RAG-2 heterodimers (3, 4), even if rare events have been reported in mice expressing only RAG-2 (5). RAG activity is directed to Ig and TR V, D, and J genes by the presence of short DNA motives composed of a conserved heptamer and a semi-conserved A/T rich nonamer separated by a largely non-conserved spacer of 12 or 23 bases, called recombination signal sequences (RSSs). V(D)J recombination always takes place between a gene flanked by a RSS with a 12 base-pair spacer (RSS-12) and a gene flanked with a RSS with a 23 base pair spacer (RSS-23). From their discovery (6), it became apparent that the level of nucleotide conservation between RSSs was rather low (7, 8). Only the three first positions of the heptamer, at the border of the gene, are conserved in almost all the known RSSs. Most of the other positions can vary. However, it was proposed a few years ago that it is not the linear sequence *per se* which is important for the ability of a RSS to support recombination, but it is the combination of certain nucleotides at different positions throughout the RSS, including the spacer (9–11). This “mutual information” model led to the development of algorithms to quantify the recombination information content (RIC) of any RSS and predict its functionality by taking into account the identity of all the nucleotides at all the positions throughout the RSS (9, 12).

The RAG recombinase first captures a single RSS-12 or RSS-23 by interaction between the nonamer binding domain (NBD) of a RAG-1 subunit and the nonamer of the RSS. A single-strand nick is then introduced at the border of the gene and the RSS, with a 3′OH radical on the gene side, and a 5′phosphorylated end on the RSS side. This complex then captures and nicks the second RSS, with a different spacer length to form the so-called paired complex. The formation of this complex catalyzes DNA cleavage by transesterification at each gene/RSS border, and results in a cleaved signal complex (CSC) containing two pairs of hairpin-sealed coding ends and blunt, 5′ phosphorylated signal ends. Coding ends are released form the CSC and subsequently opened and processed by the endonuclease Artemis activated by the DNA-dependent protein kinase (DNA-PKcs associated with the Ku heterodimer), while the signal ends remain bound to RAG in a signal end complex (SEC) [see (13) for review]. During the joining phase of V(D)J recombination, the two coding ends and the two signal ends are, respectively, ligated together by the non-homologous end joining (NHEJ) machinery. The final products of the reaction are on the one hand a coding joint (CJ), which creates a new Ig or TR gene, and on the other hand a signal joint (SJ), generally borne on episomal DNA, which “inactivates” the signal ends. CJs exhibit extensive junctional diversity due to SE processing and random polymerization of nucleotides by the terminal nucleotidyl transferase (TdT) before ligation (14, 15). This diversity is essential in the generation of a large repertoire of different antigenic receptors on lymphocytes. SJs are generally thought to be created by the perfect head-to-head ligation of the two signal ends, but they can also show limited junctional diversity (16–18).

While V(D)J recombination is essential for the protection of the organism, the programmed creation of DNA double-strand breaks (DSBs) is a very dangerous process, as DSBs represent a serious hazard for genetic stability. A mis-handling of coding or signal ends could result in their joining with fortuitous DNA ends and generate potentially oncogenic chromosomal rearrangements (19, 20). An extreme situation is for example the deletion of RAG-2 C-terminus region. This deletion results in the destabilization of the post-cleavage complex and induces a high level of genomic instability and lymphomagenesis in mice (21). In a normal situation, these deleterious outcomes are prevented because the RAG proteins protect coding and signal ends and specifically guides them toward NHEJ resolution, as a way to avoid error-prone alternative-NHEJ repair and suppress random genomic integration through homologous recombination (22). The recent elucidation of the crystallographic structure of paired complex (23–26) and SEC (4) complexes indeed shows that, in addition to the NBD-nonamer, RAG-1 and RAG-2 establish multiple contacts with numerous bases of the RSSs, including the first three bases of the heptamer and several positions in the spacer and the flanking coding region. These interactions undoubtedly participate in the protection of coding and signal ends after the cleavage step. Moreover, the assembly of the paired complex induces conformational changes resulting in a tighter, closed RAG complex with stronger RAG-1/RAG-2 interactions (24, 25). In this complex, each RAG-1 subunit, bound to the nonamer of one RSS through its NBD interacts with both RSSs, and cleavage occurs in *trans*, on the partner RSS bound to the other RAG-1 molecule. This organization, the conformational changes of the paired complex and the numerous DNA-protein contacts participate in the stabilization of the CSC and prevents the premature release and eventual illegitimate joining of coding and signal ends. In addition, the conformational changes also contribute to the exact positioning of the RSS-12 and RSS-23 in the RAG complex, so that the catalytic sites of both RAG-1 subunits are correctly positioned to nick the DNA precisely at the border of each gene and its RSS (26).

We recently performed a high throughput profiling of rearranged TRB genes present in the peripheral blood of control and irradiated CBA/Ca mice to analyze the effects of age and radiation exposure on the TR repertoire (27). More than 500,000 TRB sequences were generated during this project. A screen for rearranged TRB sequences with unusual V(D)J recombination features led to the identification of TRB genes in which germline (GL) DNA flanking the V, D, and J genes, including part or all of the RSS, was retained in their coding joint. These sequences fall into two groups: those for which this retention of germline DNA can be attributed to recombination with an upstream, alternative RSS, and those in which such RSS cannot be identified. In this second category, the presence of GL DNA most probably results from targeting errors of the RAG activity. These events also occur in human TRB and TRG genes. Although they are very rare, we believe that they may represent a threat for genomic integrity.

## Materials and Methods

### Generation of Rearranged TR Gene Repertoires

All the T cell receptor gene repertoires analyzed in this study have been generated by Adaptive Biotechnologies (Seattle, WA, USA) by high throughput sequencing following multiplex amplification of all possible V(D)J combinations from genomic DNA prepared from peripheral blood mononuclear cells (human samples), from whole peripheral blood (CBA/Ca mice) or from thymocytes and splenocytes (Balb/c and C57BL/6 mice). After sequencing, rearranged TR genes were automatically annotated (identity of the V, D, and J genes, number of nucleotides deleted from their ends, number of untemplated N nucleotides in V-D and D-J coding joints…) by the ImmunoSEQ ANALYZER software (Adaptive Biotechnologies). The generation of TRB repertoires from peripheral blood of control and irradiated CBA/Ca mice have recently been described (27). TRB gene repertoires generated from the thymus and spleen of 18-weeks old C57BL/6 and Balb/c mice were downloaded from the Adaptive Biotechnologies website, where they are provided as control datasets from normal, healthy mice (“Control Data Set of Healthy Mice and Strain Comparison,” Hamm, D. E., immuneACCESS, Adaptive Biotechnologies). Similarly, TRB and TRG gene repertoires generated from 26 donors were also obtained from the Adaptive Biotechnologies website (“Normal Human PBMC, Deep Sequencing, TCRB vs. TCRG comparison,” Robins H. and Pearson, O., immuneACCESS, Adaptive Biotechnologies).

The links to access these datasets are provided in the “Data Availability Statement” section below.

### TR Gene Sequences Processing

The coding ends of each gene in all coding joints (V-D and D-J for TRB genes, V-J for TRG genes) were analyzed individually. For each gene, we first selected all the coding joints in which this gene exhibited no nucleotide deletion from its end. From this dataset, we extracted the untemplated N nucleotides polymerized by the TdT (N region) in the coding joints. We then labeled these nucleotides according to their position, starting from the undeleted V, D, or J gene end. The frequency of each nucleotide at each position was calculated to establish a position probability matrix (PPM) of untemplated nucleotides for each undeleted gene end from all the sequences available in this selected dataset.

### Calculating Probabilities

Using the PPM established for each of the coding ends, the probability of occurrence of a given untemplated nucleotide stretch was calculated as a product of appropriate nucleotide probabilities:

$$\begin{array}{l} p=\overset{n}{\underset{i=1}{\prod }}P\left(N_{i}\right) \end{array}$$

where the lower index *i* corresponds to the position of nucleotides in PPM and *n* was equal to the length of sequence considered. For example for the heptamer sequence CACAGTG, the probability would be calculated as $p_{\left(CACAGTG\right)}=P{\left(C_{1}\right)}^{*}P{\left(A_{2}\right)}^{*}P{\left(C_{3}\right)}^{*}P{\left(A_{4}\right)}^{*}P{\left(G_{5}\right)}^{*}P{\left(T_{6}\right)}^{*}P\left(G_{7}\right)$. This equaled to the theoretical probability of finding this heptamer of interest by chance. To determine whether the nucleotide sequence was indeed created by chance a binomial test (28) was performed using the estimated probability and number of sequences meeting the conditions. The results with *p* < 0.05 were considered as statistically significant and identified the sequences where the presence a given nucleotide stretch is due to GL retention rather than to chance occurrence.

### Comparing Germline DNA Retention in Different Datasets

The frequency of occurrence of GL DNA retention in coding joints in different datasets was compared by calculating the Wilson frequency estimator and its 95% confidence interval (29).

The two-sided Kendall tau correlation test was performed to determine a relationship between the number of germline DNA retention occurrences in a given repertoire and the number of sequences in this repertoire.

To compare the GL retention lengths between TRB and TRG genes, normality of distribution in both gene groups was first checked with the Shapiro-Wilk test. Next, due to non-normal distributions, the U-Mann-Whitney test was used to verify the hypothesis on equality of median germline DNA retention length between human rearranged TRB and TRG genes.

## Results

During a screen for sequences with an unusually high level of N nucleotide additions in a dataset of rearranged TRB genes obtained from control and irradiated CBA/Ca mice, we noticed that the vast majority (246 out of 266) of the sequences with ≥25 untemplated nucleotides in the D-J joint (N1 region) were using TRBJ1-7 or, less frequently, TRBJ1-2. In these 246 sequences, those two J genes were rearranged without deletion, whereas nucleotide loss from the D gene was readily observed. This combination of a long N region at the D-J junction and the absence of exonucleolytic nibbling from the J coding end, which was found indiscriminately in sequences originating from control and irradiated mice, suggested a special processing of these genes ends during V(D)J recombination.

### Functional Cryptic RSSs in the Murine TRBJ Region

Interestingly, TRBJ1-7 is described not as a functional gene but as an “open reading frame” in the IMGT reference website (http://www.imgt.org) as it lacks the canonical Phe-Gly-X-Gly motif found in all J genes and has a non-canonical splice site precluding its expression in a TCRβ chain. A search performed for matches with the TRBJ1-7 gene sequence in the Mouse Genomic + Transcripts (Mouse G + T, updated on June 22, 2016) on the NCBI website using the BLASTN 2.8.0+ software (30) retrieved only matches to the unrearranged TRBJ1-07 sequences on the TRB locus on chromosome 6. Thus, it appears that our results report for the first time that TRBJ1-7 can be used in TRB gene rearrangement. To find out whether all rearrangements involving this gene are characterized by the presence of a long stretch of untemplated nucleotides, we retrieved from our datasets all the sequences using TRBJ1-7. Altogether, 218 sequences were found. They were all attributed 10 or more untemplated nucleotides at the D-J junction by automated annotation, compared to an average 2.58 N nt/sequence in our samples.

**Figure 1:**
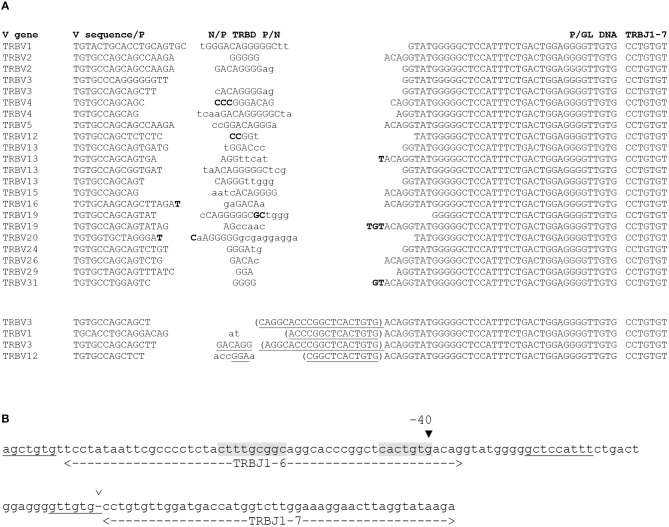
TRBJ1-7 is used in murine TRB coding joints through rearrangement of a new RSS. **(A)** Examples of murine rearranged TRB genes using TRBJ1-7. The TRBV gene used in each rearrangement is indicated on the left, and its sequence is shown from the Cys codon (TGT) until the V-D junction. The eventual P nucleotides are indicated in bold at the end of the V gene sequence. In the N/P-TRBD-P/N column, the TRBD gene is capital letters. The eventual P nucleotides are indicated in bold at the ends of the TRBD gene sequence. N nucleotides are in lowercase letters. The P/GL DNA column shows a stretch of nucleotides originally classified as N nucleotides, identical to germline DNA, with the eventual P nucleotides, in bold (see text for details). The 6 first nucleotides of the TRBJ1-7 gene are shown on the left column. The sequences in the top section are a random selection of TRB gene rearrangements having 30 to 39 nt of homology with germline DNA upstream of TRBJ1-7. The lower section shows four sequences in which the homology extends for 50–68 base upstream of TRBJ1-7. The complete collection of sequences is shown in Supplementary Table 1. **(B)** A new RSS located 40 bp upstream of the TRBJ1-7 gene, in the TRBJ1-6 gene sequence. The sequence of the TRBJ1 region encompassing TRBJ1-6 and TRBJ1-7 is shown. The boundaries of the genes are indicated by the dashed lines below the sequence. The open arrowhead above the sequence indicates the border of the TRBJ1-7 RSS described in the literature, separated from TRBJ1-7 coding sequence by a dash. Its heptamer and nonamer are underlined. The heptamer of the TRBJ1-6 gene is also underlined. The black arrowhead at position −40 indicates the position of the new RSS characterized in this study, embedded in the sequence of the TRBJ1-6 gene. Its heptamer and nonamer are shaded.

Upon alignment of these 218 sequences, it became evident that these long “N regions” were largely identical in all the rearranged genes, differing only on the D side of the D-J junction (see Figure 1A for some examples, and Supplementary Table 1 for a complete listing), a pattern suggesting that they are germline encoded rather than randomly inserted nucleotides. Indeed, a comparison with the TRBJ1 region sequence showed that these stretches of “N nucleotides” are in fact identical to the germline sequence located immediately upstream of the TRBJ1-7 gene, including its recombination signal sequence (RSS), and extending up to the TRBJ1-6 gene for the longest of them (Figure 1).

For most of the sequences (214 out of 218), this identity extends between 30 and 38 nt 5′ of the TRBJ1-7 gene. In four rearrangements, this homology is between 51 and 59 nucleotides. Seventy-two of these sequences end at position −39 relative to the beginning of the TRBJ1-7 gene. Interestingly, the sequence immediately upstream of this position constitutes a perfect RSS heptamer (CACAGTG), suggesting that this motif could anchor a functional RSS used for V(D)J recombination at this site, even though the putative corresponding nonamer motif, located 12 nt further, is less conserved when compared to the consensus sequence (GCCGCAAAG vs. ACAAAAACC, respectively). The RIC score of such a RSS, calculated on the Recombination Signal Sequence site website^1^ (12) is −48.8, suggesting that it lacks most of the mutual information required for a 12 bp RSS to be efficient, or even that it might not be functional. Interestingly, the RIC score for the RSS flanking the TRBJ1-7 coding region is almost identical (−48.7). However, we did not found any TRB gene using TRBJ1-7 through rearrangement of this RSS in our dataset.

If the −40 RSS is indeed used in V(D)J recombination, then its flanking sequence, beginning at position−39, becomes the “coding end” and should be processed as any other gene end, including the eventual insertion of P nucleotides (31) in the coding joint. Altogether, in the 70 TCRβ sequences with 39 nt of germline sequence retention, i.e., the undeleted “coding end,” we found 30 sequences with putative P nucleotides (G, GT, and GTG in 15, 10, and 5 sequences, respectively, Figure 1A and Supplementary Table 1). This finding is consistent with a mechanism involving a hairpin intermediate, and therefore suggests that the DNA breaks at this location have been generated by the RAG proteins and that this RSS is functional. To further strengthen this conclusion, we set to amplify the corresponding reciprocal signal joints (SJs) formed during the recombination of TRBJ1-7 with TRBD1 from CBA/Ca and C57BL/6 thymus DNA. PCR products of the expected size were readily obtained, cloned and sequenced. Several independent clones obtained from 2 CBA/Ca and 2 C57BL/6 mice were all found to contain a perfect fusion of the putative new RSS with the 3′TRBD1 RSS (data not shown). These findings demonstrate that a new functional RSS is located 40 nt 5′ from the TRBJ1-7 gene, embedded in the TRBJ1-6 gene coding region (Figure 1B).

**Figure 2:**
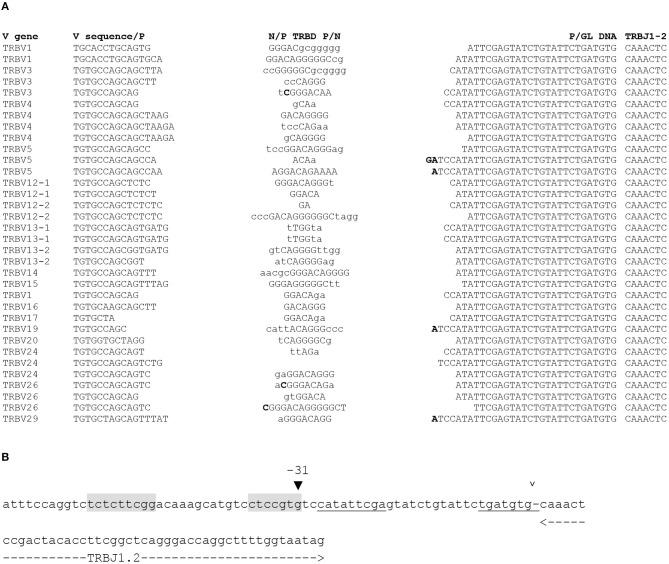
Rearrangement of the murine TRBJ1-2 through a new RSS. **(A)** Sequence of rearranged TRB genes using TRBJ1-2 through a new upstream RSS. The TRBV gene used in each rearrangement is indicated on the left, and its sequence is shown from the Cys codon (TGT) until the V-D junction. The eventual P nucleotides are indicated in bold at the end of the V gene sequence. In the N/P-TRBD-P/N column, the TRBD gene is capital letters. The eventual P nucleotides are indicated in bold at the ends of the TRBD gene sequence. N nucleotides are in lowercase letters. The P/GL DNA column shows a stretch of nucleotides originally classified as N nucleotides, identical to germline DNA, with the eventual P nucleotides, in bold (see text for details). The 6 first nucleotides of the TRBJ1-2 gene are shown on the left column. **(B)** A new RSS located 31 bp upstream of the TRBJ1-2 gene. The boundaries of the TRBJ1-2 gene are indicated by the dashed lines below the sequence. The open arrowhead above the sequence indicates the border of the TRBJ1-2 RSS described in the literature, separated from TRBJ1-2 coding sequence by a dash. Its heptamer and nonamer are underlined. The black arrowhead at position −31 indicates the position of the new RSS characterized in this study. Its heptamer and nonamer are shaded.

The second most represented gene in the group of sequences with 25 or more untemplated nucleotides was TRBJ1-2, with 30 occurrences. Here again, alignment showed that most of the nucleotides labeled as N nucleotides in these sequences by automated annotation are almost identical in all the D-J joints, and differ only on the D side. A search in our dataset retrieved 5 additional TRB rearrangements using TRBJ1-2 with a similar sequence at the D-J junction, described as containing 12 N nucleotides. The sequence of this stretch of nucleotides was found to match the sequence of the germline DNA upstream of the TRBJ1-2 gene. The identity extends from 24 to 30 nt (Figure 2A). A putative heptamer (CACGGAG) lies immediately upstream of the longest region of homology, in position −31 relative to the TRBJ1-2 gene (Figure 2B). The RIC score of a RSS anchored on this position with a 12 bp spacer and including the corresponding nonamer (CCGAAGAGA) would be −51.28, and this RSS would therefore be predicted to be non-functional. However, we could identify putative P nucleotides (A or AG) in 4 of the 5 sequences where the homology to germline sequence extends to 30 nt, i.e., at the coding ends. Here again, to show the functionality of this alternative RSS, we amplified CBA/Ca and C57BL/6 thymus DNA with primers designed to amplify the reciprocal SJs resulting from the rearrangement of TRBJ1-2 with the 3′TRBD1 RSS. Several clones were analyzed from each mouse. They were all found to contain a perfect fusion of the 3′TRBD1 and TRBJ1-2−31 RSSs, demonstrating that this RSS is used during TRB gene rearrangement. However, unlike TRBJ1-7, TRBJ1-2 is a functional gene and was found 32278 times in our dataset from 30 mice. Therefore, this new RSS located 31 bp upstream of the described TRBJ1-2 RSS is used in <0.1% of the rearranged TRB genes using TRBJ1-2, but these findings show that a functional RSS is located 31 bp upstream of the TRBJ1-2 gene. Rearrangements using this new −31 RSS result in the retention of germline sequence, including part or all of the described TRBJ1-2 RSS, in the D-J joint.

**Figure 3:**
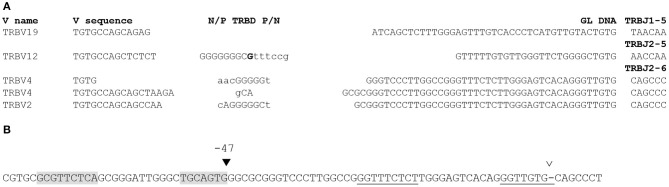
Rearranged TRB genes using TRBJ1-5, TRBJ2-5, and TRBJ2-6 including germline DNA in their D-J coding joint. **(A)** Sequence of these genes. The TRBV gene used in each rearrangement is indicated on the left, and its sequence is shown from the Cys codon (TGT) until the V-D junction. In the N/P-TRBD-P/N column, the TRBD gene is capital letters. The eventual P nucleotides are indicated in bold at the ends of the TRBD gene sequence. N nucleotides are in lowercase letters. The GL DNA column shows a stretch of nucleotides originally classified as N nucleotides, identical to germline DNA. The 6 first nucleotides of the TRBJ genes are shown on the left column. **(B)** A putative cryptic RSS located 47 bp upstream of the TRBJ2-6 gene. The open arrowhead above the sequence indicates the border of the TRBJ2-6 RSS described in the literature, separated from TRBJ2-6 coding sequence by a dash. Its heptamer and nonamer are underlined. The black arrowhead at position −47 indicates the position of the putative cryptic RSS used in the three rearranged TRB genes shown in **(A)**. Its heptamer and nonamer are shaded.

In addition to rearrangements using TRBJ1-2 or TRBJ1-7, the group of rearranged TRB gene sequences with ≥25 “untemplated nucleotides” in the D-J joint encompassed 20 other rearrangements using a diverse array of V and J genes. Upon closer analysis, 16 of these sequences were found to be either aberrant products containing two V or two J genes, probably generated during amplification, or mis-labeled sequences in which an indexing error erroneously created a long N region. One sequence was truly found to encompass 27 N nucleotides at the D-J junction (data not shown). However, in the remaining three sequences, which use TRBJ2-6, TRBJ2-5, and TRBJ1-5, the “N region” at the D-J junction was found to be identical to germline sequence upstream of the respective TRBJ genes (Figure 3). We did not found additional instances of germline sequence retention in rearrangements using TRBJ2-5 or TRBJ1-5, but two more occurrences were found for TRBJ2-6 (Figure 3). In each of these three rearrangements, a stretch of 42–46 nt is identical to the germline sequence 5′ of TRBJ2-6, including its RSS. The sequence 5′ of the longest identity stretch, starting at −47 relative to the TRBJ2-6 gene, starting with CACTGCA would anchor a RSS with a RIC score of −46.2, predicted to be weak or non-functional, and almost identical to that of the described TRBJ2-6 RSS (−45.9). Due to the low frequency of this event (found 3 times in 517,097 TRB genes), we did not attempt to amplify the signal joints generated by rearrangements using this putative RSS. Like TRBJ1-7, TRBJ2-6 is described as a pseudogene in the IMGT database, as it lacks the canonical Phe-Gly-X-Gly motif, and a proper splice site. We found it used in only 145 of the 517,097 rearranged TRB genes in our dataset. If the new −47 RSS is indeed functional, then we would have here again two RSS in close proximity with identical RIC scores used at a very different frequency, as this new −47 RSS is found in only 2% (3 out of 145) of the sequences using TRBJ2-6. In any case, collectively, these results show that significant stretches of germline DNA can be retained in coding joints by the use of alternative or cryptic, sometimes overlapping, RSS located upstream of the gene end for three different TRBJ genes in CBA/Ca mice. In two additional rearranged TRB genes, germline DNA was found retained in D-J coding joints involving TRBJ2-5 or TRBJ1-5 in the absence of the apparent use of any RSS. Prompted by these findings, we performed a systematic search for germline DNA retention in our rearranged TRB gene dataset.

### Germline Retention in TRB Gene Coding Joints in Absence of Cryptic RSS

**Figure 4:**
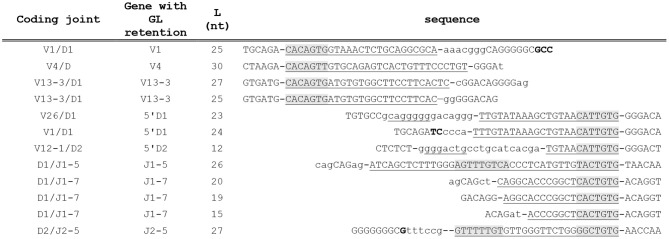
Rearranged TRB genes including germline DNA in a coding joint in CBA/Ca peripheral blood lymphocytes. The first column indicates the V and D or V and J involved in the coding joint including germline DNA retention. The gene for which GL DNA retention is observed is indicated in the second column, and the third column indicates the length (L), in nucleotides, of this stretch of GL DNA. The sequence of the coding joint is given in the last column, aligned on the left when GL DNA retention is observed from a RSS located downstream of the rearranged gene (V), and on the right when it is observed from a RSS located upstream of the rearranged gene (5′D, J). The sequence of the V-D and D-J coding joints are shown in the last column, anchored on the six last (for V) or first (for D and J) nucleotides of the genes. The stretch of GL DNA in each coding joint is underlined and the heptamer and, when present, the nonamer of the gene's RSS are overlaid in gray. When present, N nucleotides are shown in lower case letters and P nucleotides in bold letters. The D1/J1-5 and D2/J2-5 rearrangements are those reported in Figure 3. Underlined N nucleotides are identical to the sequence of TRBD genes.

We first analyzed this phenomenon for the D genes in V-D joints. TRBD genes can be identified in most of the rearrangements. The 5′ RSS of TRBD1 and TRBD2 are very similar and differ only by 3 bases. Thus, targeting our analysis at the 5′ TRBD gene RSS allows us to analyze at once most of our dataset. We first selected the rearrangements in which the intact 5′ end of the TRBD gene is identifiable. From those sequences, we selected the rearrangements with a G nucleotide immediately upstream of the D gene, then, from this selected group, those with a TG dinucleotide, then successively GTG, TGTG, TTGTG, ATTGTG and finally CATTGTG, i.e., the heptamer of TRBD1 and TRBD2 RSS. At each step, the number of selected sequences diminished drastically. Only 10 sequences had a TTGTG motif immediately upstream of an undeleted TRBD gene, and out of those, only five had the whole heptamer (CATTTGTG). Extending our search for homology beyond the heptamer, we found two rearrangements with a stretch of nine nucleotides identical to the germline sequence at the V-D junction, and one each with stretches of 12, 23, and 24 bases. It seems very unlikely that stretches of 23 and 24 nucleotides identical to germline DNA upstream of TRBD genes happened by chance; these sequences most likely represent new examples of germline DNA retention during the V(D)J recombination process. However, the situation is not so clear for those sequences with only 5, 6, 9, or 12 nucleotides of identity at the V-D junction, which could have been randomly polymerized by the TdT. Thus, to determine whether these stretches could have happened by chance, the probability of their occurrence was determined by using as reference the frequency of each nucleotide at each N nucleotide position upstream of the undeleted TRBD1 and TRBD2 genes. These calculations showed that in the case of sequences with 6, 7, and 9 nt, we could not formally rule out that they did happen by chance. Thus, only stretches of 12 nt and more could unambiguously be attributed to GL DNA retention. The same approach was reiterated for the 3′TRBD1, the 3′TRBD2 and all the TRBV and TRBJ RSSs. As for the TRBD genes, we first established the frequency of each base at each position in N nucleotides for all V and J genes. Thus, we could calculate for each of the rearranged genes the probability that a given stretch of nucleotides is germline DNA retention rather than just the random result of TdT activity. This is important as the sequence of genes ends can indeed influence their processing and therefore the sequence of the N region (32, 33). This is especially the case for the TRBV13 gene family, as these genes end in “CAC.” The corresponding P nucleotides are therefore GTG, i.e., the same 3 first nucleotides of the TRBV13 RSS. Thus, P nucleotide inclusion in the V-D coding joints would lead to an overestimation of germline DNA retention. Using this stringent approach, we could identify retention of germline DNA sequence at the V-D or the D-J joints in the absence of any recognizable RSS with a very high level of confidence in seven rearranged TRB gene sequences (Figure 4) in addition to the two TRB genes already identified, using TRBJ1-5 and TRBJ2-5 shown in Figure 3. Two of these coding joints (V26/D1 and V12-02/D2) have an unusually long N region which includes a sequence of seven nucleotides, underlined in Figure 4, that could originate from TRBD1 and TRBD2, respectively. In addition, four of the rearrangements using the −40 TRBJ1-7 RSS described above (Figure 1A) also show identity with germline DNA upstream of this new RSS. We used a slightly different approach to determine whether these sequences truly represented retention of germline DNA or were merely chance occurrences. We did not have enough sequences to establish with certainty the pattern of N nucleotide additions at the new TRBJ1-7 “coding end” resulting from the use of the −40 RSS described in the first section. Therefore, we used as reference the frequency of each nucleotide at each position from N nucleotides found in all D-J coding joints created with an undeleted J gene. This calculation showed that it was not possible to determine whether the presence of a stretch of 12 nt upstream of the −40 TRBJ1-7 RSS truly resulted from the retention of germline DNA or was a chance occurrence. However, we could conclude with a high level of confidence that in the three other sequences, the presence of 15, 19, and 20 nucleotides of homology resulted from the inclusion of germline DNA in the coding joint during the V(D)J recombination process (Figure 4). Thus, in total, we identified 12 rearranged TRB genes, found one time each, in which germline DNA is retained in a coding joint in the absence of an obvious putative RSS in our dataset of 517086 TRB gene rearrangements obtained from CBA/Ca peripheral blood lymphocytes (Figure 4). This event is clearly very rare. To ascertain that these observations did not result from a specificity of the V(D)J recombination mechanism in CBA/Ca mice, we applied the same analysis pipelines to rearranged TRB genes datasets obtained from the thymus and spleen of C57BL/6 and Balb/c mice, available as controls on the Adaptive Biotechnologies website.

**Figure 5:**
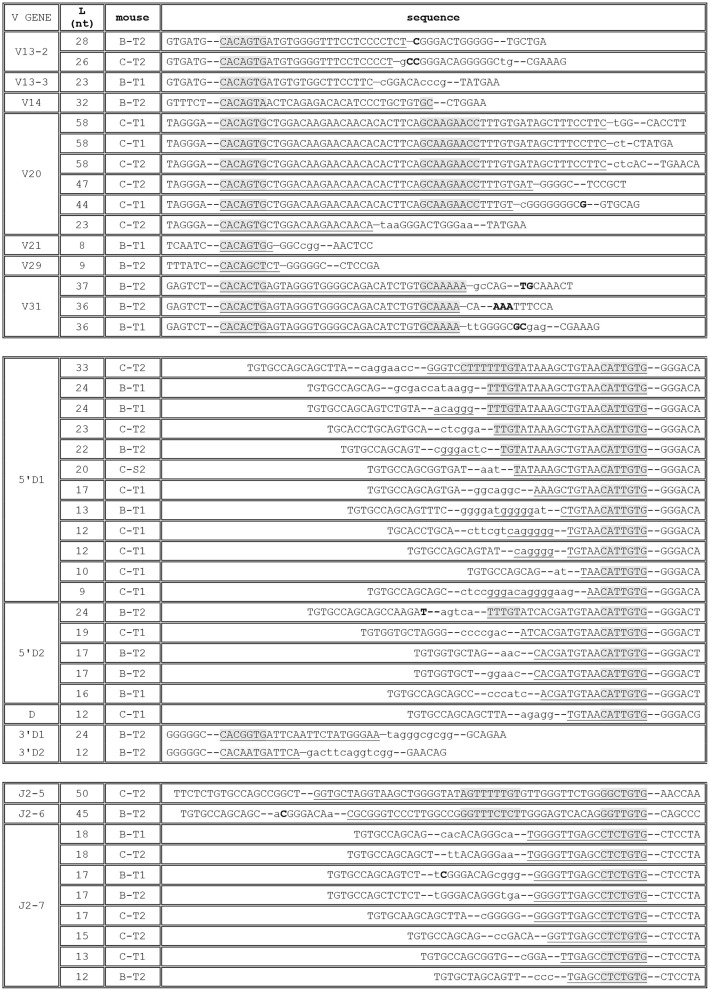
Rearranged TRB including germline DNA in a coding joint in C57BL/6 and Balb/c mice. The first column indicates the gene for which GL DNA retention is observed. The second column indicates the length (L), in nucleotides, of this stretch of GL DNA, and the third the origin of the TRB gene (C, C57BL/6; B, Balb/c; T, thymus; S, spleen; 1 or 2, the ID of the mouse). The sequence of the rearranged TR gene or of the V-D and D-J coding joints is given in the last column, aligned on the left when GL DNA retention is observed from a RSS located downstream of the rearranged gene (V, 3′D), and on the right when it is observed from a RSS located upstream of the rearranged gene (5′D, J). The sequence are anchored on the six last (for V, 3′D) or first (for 5′D and J) nucleotides of the genes. The heptamer of the RSS and, when present, the nomaner, are shaded in gray in the stretch of GL DNA in each coding joint. When present, N nucleotides are shown in lower case letters and P nucleotides in bold letters. Underlined N nucleotides are identical to the sequence of the TRBD1 gene.

**Figure 6:**
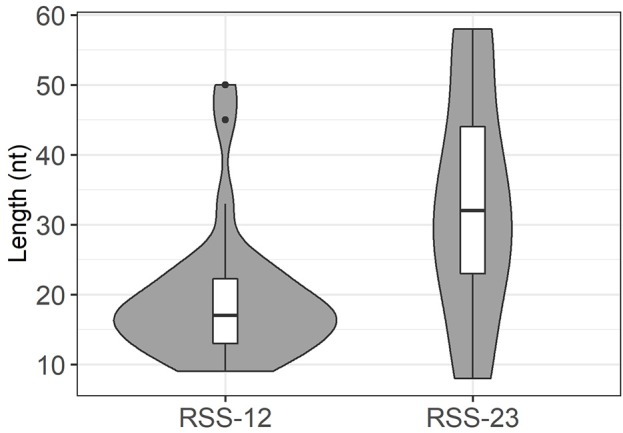
Distribution of germline DNA retention length at genes with RSS-12 and RSS-23 in murine TRB coding joints. These violin plots represent the distribution and median value of GL DNA retention length at coding ends in rearranged TRB genes in Balb/c and C57BL/6 mice. The median value is higher at genes with a RSS-23 (*p* = 4.0*10^−3^ by a U-Mann-Whitney test). Dots represent outlying values.

In these datasets, TRBJ1-7 is used in 708 rearranged TRB genes using different V genes (Supplementary Table 2). The vast majority (663) of these rearrangements are compatible with the use of the −40 RSS described in CBA/Ca mice, with deletions of up to 14 nt. In the 203 rearrangements without deletion from the TRBJ1-7 “coding end,” 75 contain 1 to 3 P nucleotides. The remaining 45 sequences include stretches of identity with germline DNA of 1–17 nt upstream of the gene's end. Of note, in 12 sequences, these stretches are longer than 12 nucleotides, i.e., most probably germline encoded. For the remaining 33 sequences, the inclusion of 1–5 nt cannot be explicitly attributed to germline retention or TdT activity. Similarly, 118 TRB genes using TRBJ1-2 potentially rearranged through the new −31 RSS described in CBA/Ca mice were also readily identified in C57BL/6 and Balb/c mice (Supplementary Table 2). Nine of the 16 sequences without any deletion of the “coding end” include 1 to 3 P nucleotides, and three include one base (G) that could originate from the heptamer. For the other 102 sequences, deletions range mostly from 1 to 9 nt. Retention of germline DNA in V-D and D-J coding joints in absence of putative cryptic RSS was formally identified at the V-D or D-J joint in 47 rearranged TRB gene sequences, 22 from Balb/c mice and 25 from C57BL/6 mice (Figure 5). These events were observed for different V genes, the 5′ and 3′ ends of the TRBD1 and TRBD2 genes, and some TRBJ genes. Depending on the gene considered, the retained sequence ranges from 8 to 58 nucleotides. Interestingly, the median length of GL DNA retained from genes with a RSS-23 is significantly bigger than that retained from genes with a RSS-12 (*p* = 0.004 by a U-Mann-Whitney test), suggesting that the mechanisms leading to GL retention are different according to the type of RSS flanking the gene (Figure 6). Of note, as observed in CBA/Ca mice, one rearranged TR gene from the thymus of a Balb/c mouse included 46 bp of DNA identical to germline DNA upstream of the TRBJ2-6 gene in its D-J coding joint. In addition, stretches of 13–27 nucleotides identical to GL DNA were also identified in 12 rearranged TRB genes using TRBJ1-7 (Supplementary Table 2). Finally, the N region of some V-D coding joints with GL retention from the 5′TRBD gene were also found to include stretches of 6–10 nucleotides identical to the sequence of TRBD1 or TRBD2. Altogether, these results largely confirm our findings in CBA/Ca mice in un-manipulated animals of two different strains. Thus, in mice, germline DNA is retained in rearranged TRB gene coding joints, either through the use of an alternative RSS located upstream of TRBJ1-7 or TRBJ1-2 genes, or more randomly, in the absence of any putative new RSS, for a larger collection of TRB genes.

### Retention of Germline DNA in Human TRB and TRG Coding Joints

We next wanted to determine whether these observations extend to V(D)J recombination in human. Here again we availed of datasets available on the Adaptive Biotechnologies website. We analyzed germline DNA retention in a collection of rearranged TRB gene repertoires obtained from 26 healthy donors. For 23 of these donors, the rearranged TRG gene repertoires were also available and analyzed.

The number of unique TRB gene identified in the different repertoires ranged from 82,020 to 4,29,846 per donor, for a total of 6,635,892 sequences. We did not find any evidence that, like for the murine TRBJ1-7 and TRBJ1-2, human TRB genes use alternative RSS. However, we readily identified germline DNA in 94 V-D or D-J coding joints. TRB sequences including germline DNA were found in 24 of the 26 donors, with 1–9 sequence per donor (Table 1). The donors in which we did not find any event are the last and the second to last with the lowest number of rearrangements. Indeed, there is a correlation (*p* = 0.0014 by a two-sided Kendall tau correlation test) between the number of unique TRB gene sequenced in the repertoire of a given donor and the number of sequences with germline DNA retention in this repertoire. This correlation suggests that germline DNA retention occurs at a similar low frequency in every individual donor. The occurrences of germline DNA retention are clearly not equally distributed among the different V, D, and J genes. The seven events identified at V gene RSS affect only two different genes: TRBV11-3 and TRBV28. These genes are functional and the RIC score value of their respective RSS indicates that they are able to support V(D)J recombination. Most of the events identified (62) occurred at the 5′RSS flanking the TRBD1 or TRBD2 genes, which are by far the most used genes in these datasets. Like in mice, stretches of 6–8 nucleotides identical to the TRBD1 could be found in the “N region” of V-D coding joints with GL retention from the 5′TRBD1 gene (underlined N nucleotides in Supplementary Table 3). Retention of germline DNA at the TRBJ gene end was found in 24 sequences for eight different J genes, three from the TRBJ1 cluster and five from the TRBJ2 cluster. Thus, as in mice, germline DNA can be retained in V-D and D-J coding joints during the rearrangement of human TRB genes, from RSS-12 and RSS-23, in absence of any putative RSS.

**Table 1:** Summary of GL DNA retention in human TRB genes.

**Donor ID**	**nb of unique TRB sequences**	**nb with GL retention**	**Gene (nb of GL nucleotides)**
HIP01181	102017	2	TRBJ1-3 (22), TRBJ1-6 (25)
HIP05941	267329	4	TRBD1 (11,11,17), TRBJ2-1 (21)
HIP13244	234627	2	TRBD1 (14,12)
HIP13309	429849	5	TRBV28-1 (11), TRBD1 (27,16,11), TRBD2 (14)
HIP13350	245731	3	TRBD1 (13,12), TRBD2 (14)
HIP13376	411273	9	TRBD1 (18,17,15,14,11,11), TRBJ1-6 (32), TRBJ2-4 (19,14)
HIP13511	292766	8	TRBD1 (17,15,12,11,11), TRBD2 (12), TRBJ1-6 (28), TRBJ2-7 (12)
HIP13741	378826	3	TRBD1 (13), TRBD2 (28), TRBJ2-3 (11)
HIP13749	289975	5	TRBV28.1 (10,10), TRBD1 (19,13,11)
HIP13769	236598	1	TRBD1 (14)
HIP13803	277626	5	TRBD1 (23), TRBJ1-2 (22), TRBJ2-4 (37,17), TRBJ2-6 (11)
HIP13806	157436	3	TRBV28-1 (10), TRBD1 (17), TRBJ1-6 (28)
HIP13823	190464	2	TRBD1 (20,15)
HIP13831	149119	1	TRBD1 (13)
HIP13939	267205	5	TRBD1 (32,11), TRBD2 (14,10), TRBJ1-5 (18)
HIP13951	409325	7	TRBV11-3 (8), TRBD1 (18,14,11), TRBD2 (15), TRBJ1-5 (20), TRBJ2-4 (11)
HIP13958	306691	3	TRBD1 (16,11), TRBD2 (19)
HIP13967	220861	5	TRBD1 (18,13,11), TRBJ2-1 (21), TRBJ2-7 (12)
HIP13975	363702	3	TRBD1 (14), TRBJ1.6 (28,19)
HIP13981	213139	1	TRBD1 (16)
HIP13992	105882	0	
HIP14064	310173	1	TRBD2 (10)
HIP14121	258248	7	TRBV11.3 (14), TRBV28-1 (10), TRBD1 (32,17,14,12), TRBJ2-6 (28)
HIP14152	82020	0	
HIP14209	174386	2	TRBD1 (26,11)
HIP14213	260624	6	TRBD1 (16,15,14,11), TRBJ1-5 (20,20)
All	6635892	93	Average nb of GL nucleotide/seq: 16.33

**Figure 7:**
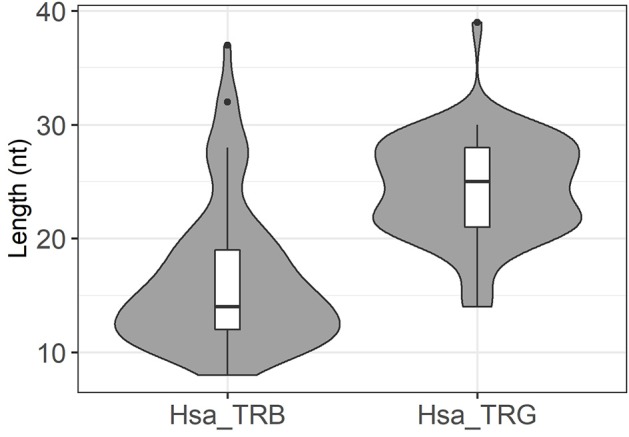
Distribution of germline DNA retention length in coding joints in human rearranged TRB and TRG genes. These violin plots represent the distribution and median value of GL DNA retention length at coding ends in rearranged human TRB and TRG genes. The median value of germline DNA retention length is higher in TRG coding joints (*p* < 10^−9^ by a U-Mann-Whitney test). Dots represent outlying values.

Finally, we extended our analysis to the rearranged TRG gene repertoires determined in parallel of the TRB gene repertoire for 23 of the 26 donors. One of the repertoires included only 677 unique sequences and was excluded from further analysis. The remaining 22 datasets were comprised of 99,534–4,44,612 sequences per donor, for a total of 6,284,293 rearranged TRG genes. We identified 67 instances of germline DNA retention in coding joints, essentially from the V gene end (Table 2). Nine of the 14 TRGV genes and one TRGJ gene were affected, with retention of 9–39 bases. They were found in all donors (1–9 events per donor), and here again we observed a correlation between the number of sequences in the repertoire and the number of germline DNA retention occurrences in this repertoire (*p* = 0.03, by a two-sided Kendall tau correlation test). Of note, germline DNA retention happens in TRG genes in the two donors for whom we could not find any in rearranged TRB genes. Thus, this phenomenon takes place in all of the 26 donors analyzed. For eight of the V genes, retention occurs in the absence of putative alternative RSS. The situation is less clear for TRGVB. In the IMGT database, this gene is described as a pseudogene because of a stop codon and several frameshifts. In addition, examination of its RSS reveals a low RIC score of −69.14, suggesting that it is not functional. However, TRGVB is used in 45 rearranged TRG genes (Supplementary Table 3). One to six rearranged TRGVB genes per donor were found in 18 of the 22 donors. Thirty-four of these rearranged genes display stretches of 11–25 potentially germline nucleotides at the undeleted TRGVB coding end. Two additional putative RSSs can be identified 15 and 27 bases downstream of TRGVB gene, anchored by CACACTG and CACAACA, respectively. The first one has a RIC score value of −57.28, just above the threshold for functionality, whereas the RIC score for the second one is of −74.24, indicating that it should be non-functional. Given the usual exonucleolytic nibbling of coding ends during V(D)J recombination, coding joints with 27–16 nt of germline DNA could originate from recombination of the −27 putative RSS, those with 15–11 germline-encoded nucleotides from recombination of the −15 RSS and those without germline DNA retention from recombination of the described TRGVB RSS. Of note, six of the 11 TRGVB genes in this last group exhibit 1 to 5 P nucleotides from TRGVB end. Thus, TRGVB is rearranged with TRGJ genes at very low levels in most of the donors either from the RSS flanking its coding sequence or from other low activity putative cryptic RSS. Given the rarity of these events *in vivo*, we did not investigate further the functionality of these putative RSSs. In any case, irrespective of the exact mechanisms involved, these results show that the retention of germline DNA in coding joints is not a specificity of TRB gene rearrangement in man, as it also occurs during V(D)J recombination of TRG genes in all of the donors analyzed here. Interestingly, the average length of GL DNA retention is bigger in TRG genes (24.6 nt/seq) than in TRB genes (16.3 nt/seq) (Tables 1, 2) and the median length of GL DNA retention at TRG genes is bigger than at TRB genes (Figure 7, *p* < 10^−9^ by a U-Mann-Whitney test). Considering that the vast majority of the events identified for TRG genes concern TRGV genes, with RSS-23, whereas for TRB genes they concern the 5′ end of the TRBD genes, flanked by RSS-12, this difference is reminiscent of our earlier finding in mice of a different processing of genes flanked by RSS with different spacer length (Figure 6).

**Table 2:** Summary of GL DNA retention in human TRG genes (except TRGVB).

**Donor ID**	**nb of unique TRG sequences**	**nb with GL retention**	**Gene (nb of GL nucleotides)**
HIP01181	127569	2	TRGV2-1 (23), TRGV8-1 (25)
HIP05941	325934	5	TRGV6-1 (21), TRGV11-1 (29,27,20,19)
HIP13244	274413	4	TRGV2-1 (22), TRGV11-1 (29,29,27)
HIP13309	412483	9	TRGV7-1 (21), TRGV8-1 (23,21), TRGV11-1 (29,29,29,26,26,23)
HIP13376	406349	3	TRGV11-1 (26,26,21)
HIP13511	327805	4	TCRGV2-1 (26), TRGV10-1 (23), TRGV11-1 (29,22)
HIP13741	426644	3	TRGV8-1 (21), TRGV11-1 (29,27)
HIP13769	272913	4	TRGV2-1 (21,14), TRGV11-1 (29,19)
HIP13803	329253	1	TRGV8-1 (21)
HIP13806	212234	1	TRGV11-1 (26)
HIP13823	219204	2	TRGV10-1 (24), TRGV11-1 (26)
HIP13831	172321	2	TRVG2-1 (23), TRGV11-1 (29)
HIP13939	311247	2	TRGV5P-1 (23), TRGV11-1 (21)
HIP13951	444612	3	TRGV8-1 (17), TRGV11-1 (29,25)
HIP13958	346863	2	TRGV2-1 (20), TRGV11-1 (29)
HIP13975	677		Outlier, not considered
HIP13981	252444	2	TRGV11-1 (30), TRGJP1 (14)
HIP13992	127238	3	TRGV7-1 (22), TRGV11-1 (29,26)
HIP14064	374918	4	TRGV11 (29,26,24,21)
HIP14121	291792	3	TRGV10-1 (27), TRGV11-1 (28,24)
HIP14152	99534	1	TRGV10-1 (23)
HIP14209	213320	1	TRGV11-1 (29)
HIP14213	314526	4	TRGV2-1 (39), TRGV7-1 (21), TRGV9-1 (25), TRGV11-1 (19)
All	6284293	65	Average nb of GL nucleotide/seq: 24.6

## Discussion

The analysis of rare, unusual rearranged TRB gene sequences led to the unambiguous identification of the retention of germline DNA flanking the TRB and TRG V, D and J genes in V-D, D-J, and V-J coding joints. This DNA should normally have been removed from the V, D, or J gene coding ends during the V(D)J recombination, if the RAG recombinase had, as expected, introduced a nick exactly at the border of the genes and their described RSSs. The low frequency of these events does not have any consequence on the diversity of the functional expressed TR gene repertoire in human and mice. However, we think their identification provides new information that will help shed a new light on the precise mechanisms at play during normal and pathogenic V(D)J recombination *in vivo*. We found two categories of sequences with GL DNA retention.

In the first category, GL DNA is retained in coding joints because the RAG recombinase used an upstream alternative or cryptic RSS. These events, identified for the rearrangement of TRBJ1-2, TRBJ1-7 and, most probably, TRBJ2-6, lead to the retention of germline DNA sequence in the D-J coding joints, including all or part of the RSS lying immediately upstream of the J gene. TRBJ1-7 is described as an ORF and was never described rearranged. Here, we found that it can be used for V(D)J recombination in a collection of rearranged TRB genes. However, V(D)J recombination was mediated not through the use of the described RSS flanking the TRBJ1-7 coding sequence, but through the use of an alternative RSS located 39 bases upstream. In fact, this new alternative RSS is the only one used for TRBJ1-7 rearrangement in CBA/Ca, despite the fact that it has the same RIC score than the RSS described at the border of the TRBJ1-7 gene. These two RSSs are largely dissimilar and share only a few bases of homology: they have identical nucleotides at only nine positions throughout the RSS sequence, including the very well conserved CACA nucleotides anchoring the heptamer. Thus, the same RIC score can describe two very different RSSs, one functional and used, even if rarely, and one not functional and not used at all. In contrast, for the TRBJ1-2 gene, the new and published RSS have very different RIC score values, −51.28 and −35.64, respectively. In this case, the difference in RIC scores is reflected in the difference in rearrangement frequency, and this new RSS can probably be described as “cryptic.” Interestingly, the sequence of this new RSS heptamer overlaps with that of the nonamer of the published TRBJ1-2 RSS. Thus, most of the times, the RAG proteins are able to discriminate between two overlapping functional RSSs of different “fitness” and select the best in accessible chromatin during T lymphocyte development. This finding illustrates a new aspect of the exquisite specificity of RAG1/RAG2 docking and activity in a physiological setting. These observations will surely be of help in trying to understand the determinants of RSS functionality and refine RIC score calculation.

**Figure 8:**
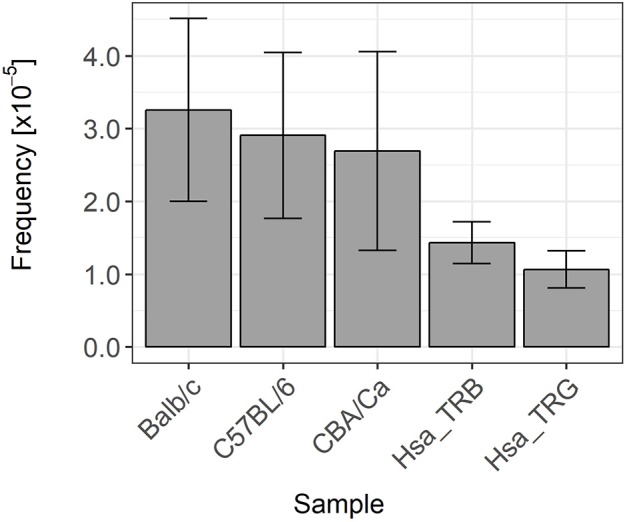
Germline DNA retention frequency in murine and human TRB and TRG coding joints. These histograms represents the Wilson frequency estimators and 95% confidence intervals of the frequency of occurrence of germline DNA retention in coding joints in the different datasets analyzed in this study.

In the second group of coding joints exhibiting GL DNA retention, we could not identify the involvement of any alternative or cryptic RSS mediating proper V(D)J recombination but at an improper site. The presence of GL DNA in these coding joints most probably denotes a mis-targeting of the RAG cleavage activity during V(D)J recombination. These errors are clearly very rare, with a frequency ranging from 0.003% in Balb/c mice to about 0.001% in rearranged human TRB and TRG genes (Figure 8). It must be pointed out that these estimations represent a minimum value, as we included in our calculations only instances were statistical analysis showed unambiguously that the considered stretches of GL DNA could not have happened by chance. In numerous cases, we identified sequence with stretches of nucleotides identical to germline DNA too short to be statistically attributed to errors in cleavage or to the activity of the TdT in individual sequences. However, if we took into consideration the number of these occurrences, then it became highly likely that these events were not random. This was, for example, the case for two different murine TRB genes including a stretch of nine bases identical to GL DNA at the 5′ end of the TRBD2 gene. The possibility that one sequence had been generated by chance cannot be excluded, but the fact that we found two rearranged TRB genes with the same “GL” sequence in our dataset becomes highly significant. A lot of similar examples were found in our datasets, suggesting that these events are actually more frequent than reported in this paper.

In our murine datasets, the distribution of length of the GL DNA retention stretches is clearly different at genes flanked by RSS-12 and genes flanked by RSS-23. This observation suggests that the mechanisms generating these errors have different constraints on both types of genes. It may be that they relate to the physical forces operating on the DNA stretches bearing the RSS in the RAG complex. Crystal structures showed that RSSs have to be bent or “kinked” in order to be accommodated within the complex (23). HMGB1 most probably plays an important role in this positioning (34). It may be that these errors are generated in complexes where HMGB1 is not properly positioned, or that not enough or too many HMGB1 molecules participate in the complex, resulting in an inadequate location of the heptamer in relation to the RAG-1 catalytic site. As the RSS nonamer is not highly conserved (7, 8), it may also be that the binding of RAG-1 NBD is not 100% precise and can be off for a few bases from the optimal position, not as tight as expected. The closing of the RAG complex upon capture of the second RSS (25) may then induce a “slippage” of the complex, leading to a mis-positioning and the introduction of the nick at a wrong base. We can only speculate at that time, but our observations may be useful in refining the RAG/DNA complex model, by trying to model DNA locations resulting in such errors. However, whatever the exact mechanism(s) leading to retention of GL DNA in coding joint, their frequency is clearly different at genes flanked by RSS-12 and RSS-23 in mice, and also in human as suggested by the differences observed at TRG and TRB genes. These differences suggest that TR genes are differently recognized and/or processed at the time of nicking according to the structure of their RSS, despite the apparent symmetry of the two RAG-1/RAG-2 heterodimers in the RAG complex. Finally, we also noted that the frequency of GL retention is lower in human than in murine rearranged TR genes. This observation suggests that the precision of RAG cleavage activity is different in human and murine cells, maybe due to differences in RAG protein sequence and/or structure, which could promote a better control of positioning or stronger interactions in human thymocytes.

The events described here, although unusual, result from *bona-fide* V(D)J recombination of chromatinized TR genes in accessible conformation, performed *in vivo* in physiological conditions in developing thymoytes in human and mice. Despite their very low frequency, we believe that they represent a potential threat for genetic stability and may promote lymphomagenesis. DSBs generated during antigen receptor gene assembly are a source of DNA ends that can be involved in illegitimate genetic rearrangements (19, 20). The development of T-acute lymphoid leukemia (T-ALL) is for example thought to be initiated by V(D)J recombination-mediated oncogenic translocations involving TR genes (35). One of the current models for these deleterious illegitimate rearrangements posits that a “foreign” (non-TR) fortuitous DNA end can invade the post-cleavage complex during V(D)J recombination, and be ligated to a signal or coding end to generate an oncogenic chromosomal rearrangement (20). Our results now suggest a different possibility. The introduction of a DSB away from the border of gene during V(D)J recombination will result in the generation of truncated SE, with deletions of up to 37 bases (for human TRB genes), including part or all of the RSS. In the SEC, the RAG proteins establish numerous contacts with the heptamer and the beginning of the spacer region (24, 25). This tight association of signal ends with the RAG complex is essential to “shepherd” them toward resolution by NHEJ, as a way to neutralize these DNA ends and prevent their random re-introduction in the genome (22). If part or all of these nucleotides are deleted from the signal ends because of GL retention, these contacts will not be established. The overall interactions between the DNA ends and the RAG complex will be weaker, and consequently the post-cleavage SEC will be less stable, allowing the escape of the truncated signal ends from the V(D)J recombination center. These wandering signal ends will then be available to be ligated with any DNA end generated by fortuitous DSB elsewhere in the genome, without the need for this end to invade the CSC or the SEC. In support of this hypothesis, an example of such event can be found in the Supplementary Figure S5 accompanying the study of Le Noir et al. (35), where one of the partners of the translocation involving two chromosomes 7 identified in a T-ALL (T-ALL 346) is a 5′TRBD1 SE truncated of 14 bases. Thus, while the model of V(D)J recombination-mediated translocation is not new, our work identifies for the first time V(D)J recombination errors that may lead to such translocations, generated *in vivo* during TR gene rearrangement. Alternatively, the deleted signal ends unprotected or less protected by the RAG complex, can also be re-integrated in the genome through homologous recombination if NHEJ does not timely ligate them to form a signal joint, promoting genetic instability (22). Therefore, although they are very rare, errors in the targeting of RAG activity may initiate different mechanisms resulting in genetic instability and potentially oncogenic illegitimate rearrangement, and have detrimental consequences on health.

Finally, we also identified a putative signature of illegitimate V(D)J recombination in a limited number of murine and human TRB genes. The presence of two TRBD genes in tandem (Figures 4, 5, Supplementary Table 3) indeed raises the possibility that these rearrangements have been generated by inter-chromosomal V(D)J recombination, a very deleterious event. It has been described that the RAG recombinase brings antigen-receptor loci on both alleles in close proximity in developing lymphocytes, but recombination is limited to only one allele as introduction of DSB on the first locus activates ATM-dependent mechanisms to suppress recombination of the locus borne on the second allele (36, 37). Our results now suggest that this mechanism, aimed at preventing inter-chromosomal V(D)J recombination-mediated translocation is not failsafe, neither in human nor in mice. Interestingly, such bi-allelic rearrangements were identified in rearranged TR sequences exhibiting GL DNA retention. This coincidence of two V(D)J recombination errors in a single sequence suggests that the mechanisms aimed at preserving genetic integrity are less efficient in certain developing thymocytes. It will be interesting to determine whether the frequency of rearranged TR genes with GL DNA retention in a coding joint and/or with TRBD genes rearranged in tandem is increased in patients suffering from lymphoid neoplasms.

In conclusion, our systematic analysis of errors generated during V(D)J recombination in datasets obtained by high throughput profiling or rearranged TRB genes in mice and men identified two mechanisms leading to the retention of germline DNA in coding joints. The use of alternative RSS embedded in TR genes, sometimes overlapping with other RSS, is interesting from a mechanistic point of view and will probably prove useful in refining algorithms aimed at predicting RSS functionality. These events, identified only in mice, do not threaten genome integrity. In contrast, we believe that the second type of errors identified in mice and men represents a danger for genomic stability. The retention in coding joints of germline DNA, including part or all of the RSS, will result in less stable RAG-signal ends complexes. Consequently, these signal ends may escape and become available for a random re-integration in the genome, potentially resulting in an oncogenic translocation. These errors are very rare, but so is the development of V(D)J-mediated lymphoid neoplasm. The identification from the literature of a translocation sequence “compatible” with this model, found in a case of T-ALL, lends support to our model.

## Data Availability Statement

The datasets analyzed in this study are all accessible on the Adaptive Biotechnologies website, after free registration: CBA/Ca TRB repertoires: https://clients.adaptivebiotech.com/pub/candeias-2019-mouse. C57BL/6 and Balb/c TRB repertoires: https://doi.org/10.21417/ADPT2015MC. Human TRB and TRG repertoires: https://doi.org/10.21417/B7SG6T.

## Ethics Statement

The animal study was reviewed and approved by the Home Office and the animal welfare and ethical review body of the Centre for Radiation, Chemical and Environmental Hazards Public Health England Chilton. All animal procedures conformed to the United Kingdom Animals (Scientific Procedures) Act 1986, Amendment Regulations 2012.

## Author Contributions

CB obtained the CBA/ca TRB repertoires. SC and JM identified and characterized unusual coding joints. SK and SC characterized SJ from alternative RSSs. JP and JM were responsible for the design of the data analysis pipeline, performing the comparison study, and the development and implementation of the data analysis algorithms. JM, JP, and SC analyzed the data. SC, JM, JP, and CB wrote of the manuscript. All authors read and approved the final manuscript.

### Conflict of Interest

The authors declare that the research was conducted in the absence of any commercial or financial relationships that could be construed as a potential conflict of interest.

## Footnotes

^1^https://www.itb.cnr.it/rss/index.html

## Abbreviations

TR: T cell receptor
RAG: recombination activating gene
RSS: recombination signal sequence
CSC: cleaved signal complex
SEC: signal end complex
TdT: terminal nucleotidyl transferase.
