# Supplementary material for: Germline DNA Retention in Murine and Human Rearranged T Cell Receptor Gene Coding Joints: Alternative Recombination Signal Sequences and V(D)J Recombinase Errors
Source: Front Immunol. 2019 Nov 8;10:2637. doi: 10.3389/fimmu.2019.02637 (PMC6857471; doi:10.3389/fimmu.2019.02637)
Supplement: Supplementary file 1 [file Table_1.docx]

**Supplemental Table 1 : Rearranged TRB genes using TRBJ1-7 with the -40 RSS in CBA/Ca mice**

The TRBV gene used in each rearrangement is indicated on the left, and its sequence is shown from the Cys codon (TGT) until the V-D junction. The eventual P nucleotides are indicated in bold at the end of the V gene sequence. In the N/P-TRBD-P/N column, the TRBD gene is capital letters. The eventual P nucleotides are indicated in bold at the ends of the TRBD gene sequence. N nucleotides are in lowercase letters. The P/GL DNA column shows a stretch of nucleotides originally classified as N nucleotides, identical to germline DNA, with the eventual P nucleotides, in bold (see text for details). The 6 first nucleotides of the TRBJ1-7 gene are shown on the left column.

vGeneName nucleotide

TRBV1 TGTACTGCACCTGCAGTGC tGGGACAGGGGGCtt GTATGGGGGCTCCATTTCTGACTGGAGGGGTTGTG CCTGTGT

TRBV1 TGTACTGCACCTGCAGTG tcACAG **T**ACAGGTATGGGGGCTCCATTTCTGACTGGAGGGGTTGTG CCTGTGT

TRBV1 TGTACTGCACCTGCAG gcGACAGGGag GGTATGGGGGCTCCATTTCTGACTGGAGGGGTTGTG CCTGTGT

TRBV1 TGCACCTGCAGTG gccCCCAGGG ACAGGTATGGGGGCTCCATTTCTGACTGGAGGGGTTGTG CCTGTGT

TRBV1 TGCACCTGCAGTGCAG aagcACAGGGaga AGGTATGGGGGCTCCATTTCTGACTGGAGGGGTTGTG CCTGTGT

TRBV1 TGCACCTGCAGTGCAG GACAGGGaggaaag GGTATGGGGGCTCCATTTCTGACTGGAGGGGTTGTG CCTGTGT

TRBV1 TGCACCTGCAGTGCAG ccaCCGGGACAGGGa ACAGGTATGGGGGCTCCATTTCTGACTGGAGGGGTTGTG CCTGTGT

TRBV1 TGCACCTGCAGTGC CAa ACAGGTATGGGGGCTCCATTTCTGACTGGAGGGGTTGTG CCTGTGT

TRBV1 TGCACCTGCAGTG GGACAGGGc CAGGTATGGGGGCTCCATTTCTGACTGGAGGGGTTGTG CCTGTGT

TRBV1 TGCACCTGCAGTGCAG atta GTATGGGGGCTCCATTTCTGACTGGAGGGGTTGTG CCTGTGT

TRBV2 TGTGCCAGCAGCCAAGA GGGGG ACAGGTATGGGGGCTCCATTTCTGACTGGAGGGGTTGTG CCTGTGT

TRBV2 TGTGCCAGCAGCCAAGA ggGGGAgtg GGTATGGGGGCTCCATTTCTGACTGGAGGGGTTGTG CCTGTGT

TRBV2 TGTGCCAGCAGCCAA AGGGttggg GGTATGGGGGCTCCATTTCTGACTGGAGGGGTTGTG CCTGTGT

TRBV2 TGTGCCAGCAGCC CA GTATGGGGGCTCCATTTCTGACTGGAGGGGTTGTG CCTGTGT

TRBV2 TGTGCCAGCAGCCAA ACAGGGa TATGGGGGCTCCATTTCTGACTGGAGGGGTTGTG CCTGTGT

TRBV2 TGTGCCAGCAGCCAAGA CA ATGGGGGCTCCATTTCTGACTGGAGGGGTTGTG CCTGTGT

TRBV2 TGTGCCAGC caaGACAGGGat GGTATGGGGGCTCCATTTCTGACTGGAGGGGTTGTG CCTGTGT

TRBV2 TGTGCCAGCAGCCAAG gggggCAGGGGGCag GGTATGGGGGCTCCATTTCTGACTGGAGGGGTTGTG CCTGTGT

TRBV2 TGTGCCAGCAGCCAA agggggCAGGGGGCag GGTATGGGGGCTCCATTTCTGACTGGAGGGGTTGTG CCTGTGT

TRBV2 TGTGCCAGCAGCC tCAGGGaggg GGTATGGGGGCTCCATTTCTGACTGGAGGGGTTGTG CCTGTGT

TRBV2 TGTGCCAGCAGCCAAGA GACAGGGGag GGTATGGGGGCTCCATTTCTGACTGGAGGGGTTGTG CCTGTGT

TRBV2 TGTGCCAGCAGCCAA a GTATGGGGGCTCCATTTCTGACTGGAGGGGTTGTG CCTGTGT

TRBV2 TGTGCCAGCAGCCAAGA**TC** cgcCAGGGGGC**GC**t AGGTATGGGGGCTCCATTTCTGACTGGAGGGGTTGTG CCTGTGT

TRBV2 TGTGCCAGCAGCCAAGA**TC** cgcCAGGGGGC**GC**t AGGTATGGGGGCTCCATTTCTGACTGGAGGGGTTGTG CCTGTGT

TRBV2 TGTGCCAGCAGC tcaACAGGGag AGGTATGGGGGCTCCATTTCTGACTGGAGGGGTTGTG CCTGTGT

TRBV2 TGTGCCAGCAGCCAAGA GACAGGG ACAGGTATGGGGGCTCCATTTCTGACTGGAGGGGTTGTG CCTGTGT

TRBV3 TGTG tacttctgtgcCAGcagctaaa GTATGGGGGCTCCATTTCTGACTGGAGGGGTTGTG CCTGTGT

TRBV3 TGTGCCAGCAGC cc**CC**GGGACAGag AGGTATGGGGGCTCCATTTCTGACTGGAGGGGTTGTG CCTGTGT

TRBV3 TGTGCCAGCAGCTTAG tGGGACAGGGGagg AGGTATGGGGGCTCCATTTCTGACTGGAGGGGTTGTG CCTGTGT

TRBV3 TGTGCCAGCAGCTTAG GGC GGGGGCTCCATTTCTGACTGGAGGGGTTGTG CCTGTGT

TRBV3 TGTGCCCAGGGGGGTT GGTATGGGGGCTCCATTTCTGACTGGAGGGGTTGTG CCTGTGT

TRBV3 TGTGCCAGCAGCTTAG gggtGGGGGCcc ACAGGTATGGGGGCTCCATTTCTGACTGGAGGGGTTGTG CCTGTGT

TRBV3 TGTGCCAGCAGTG ggAGGGt GGTATGGGGGCTCCATTTCTGACTGGAGGGGTTGTG CCTGTGT

TRBV3 TGTGCCAGCAGCT CAGGGGagg AGGTATGGGGGCTCCATTTCTGACTGGAGGGGTTGTG CCTGTGT

TRBV3 TGTGCCAGCAGCTTA cgCAGGGGGC GGGGGCTCCATTTCTGACTGGAGGGGTTGTG CCTGTGT

TRBV3 TGTGCCAGCAGC ccACAGGGtgg GGTATGGGGGCTCCATTTCTGACTGGAGGGGTTGTG CCTGTGT

TRBV3 TGTGCCAGCAGCTTA AGGGGGt GGTATGGGGGCTCCATTTCTGACTGGAGGGGTTGTG CCTGTGT

TRBV3 TGTGCCAGCAGC cccgGGGGGGGCcgg AGGTATGGGGGCTCCATTTCTGACTGGAGGGGTTGTG CCTGTGT

TRBV3 TGTGCCAGCAGC cccGGGGGGGCcgg AGGTATGGGGGCTCCATTTCTGACTGGAGGGGTTGTG CCTGTGT

TRBV3 TGTGCCAGCAGC cccGAgtag GGTATGGGGGCTCCATTTCTGACTGGAGGGGTTGTG CCTGTGT

TRBV3 TGTGCCAGCAGCTT cACAGGGGag GGTATGGGGGCTCCATTTCTGACTGGAGGGGTTGTG CCTGTGT

TRBV3 TGTGCCAGCAGC gtcCCGGGACAGGGGa AGGTATGGGGGCTCCATTTCTGACTGGAGGGGTTGTG CCTGTGT

TRBV3 TGTGCCAGCAGCT cGGACAGa ACAGGTATGGGGGCTCCATTTCTGACTGGAGGGGTTGTG CCTGTGT

TRBV4 TGTGCC cccaGACAGGGc GGATGGGGGCTCCATTTCTGACTGGAGGGGTTGTG CCTGTGT

TRBV4 TGTGCCAGCAGC GACAGGGGGCgct TGGGGGCTCCATTTCTGACTGGAGGGGTTGTG CCTGTGT

TRBV4 TGTGCCAGCAGC cGACAGGGGAggg ACAGGTATGGGGGCTCCATTTCTGACTGGAGGGGTTGTG CCTGTGT

TRBV4 TGTGCCAGCAGCTAAGA GG ACAGGTATGGGGGCTCCATTTCTGACTGGAGGGGTTGTG CCTGTGT

TRBV4 TGTGCCAGCAGC cctGGACTc **T**ACAGGTATGGGGGCTCCATTTCTGACTGGAGGGGTTGTG CCTGTGT

TRBV4 TGTGCCAGCAGCT cGGACAG ACAGGTATGGGGGCTCCATTTCTGACTGGAGGGGTTGTG CCTGTGT

TRBV4 TGTGCCAGCAGCT tAGGGGGg **GT**ACAGGTATGGGGGCTCCATTTCTGACTGGAGGGGTTGTG CCTGTGT

TRBV4 TGTGCCAGCAGC **CCC**GGGACAG CAGGTATGGGGGCTCCATTTCTGACTGGAGGGGTTGTG CCTGTGT

TRBV4 TGTGCCAGCAGC aactGACAGGGctg GGTATGGGGGCTCCATTTCTGACTGGAGGGGTTGTG CCTGTGT

TRBV4 TGTGCCAGCAGCTAAG tc GGTATGGGGGCTCCATTTCTGACTGGAGGGGTTGTG CCTGTGT

TRBV4 TGTGCCAGCAGC aGGACAGaaaaa **GT**ACAGGTATGGGGGCTCCATTTCTGACTGGAGGGGTTGTG CCTGTGT

TRBV4 TGTGCCAGCAGC cccccGGGGGGt AGGTATGGGGGCTCCATTTCTGACTGGAGGGGTTGTG CCTGTGT

TRBV4 TGTGCCAGCAGC cGACAGGGGGCt GGTATGGGGGCTCCATTTCTGACTGGAGGGGTTGTG CCTGTGT

TRBV4 TGTGCCAGCAGC cGACAGGG CAGGTATGGGGGCTCCATTTCTGACTGGAGGGGTTGTG CCTGTGT

TRBV4 TGTGCCAGCAGCTAAGA**TC** GGGGGCgca AGGTATGGGGGCTCCATTTCTGACTGGAGGGGTTGTG CCTGTGT

TRBV4 TGTGCCAGCAGCTA aggGGGACAGGGGGCta GTATGGGGGCTCCATTTCTGACTGGAGGGGTTGTG CCTGTGT

TRBV4 TGTGCCAGCAGC cGACAGGGGGCtg GGTATGGGGGCTCCATTTCTGACTGGAGGGGTTGTG CCTGTGT

TRBV4 TGTGCCAGCAG tcaaGACAGGGGGCta AGGTATGGGGGCTCCATTTCTGACTGGAGGGGTTGTG CCTGTGT

TRBV4 TGTGCCAGCAG atcGACAGGGGG CAGGTATGGGGGCTCCATTTCTGACTGGAGGGGTTGTG CCTGTGT

TRBV5 TGTGCCCTCGGGACAA ACAGGTATGGGGGCTCCATTTCTGACTGGAGGGGTTGTG CCTGTGT

TRBV5 TGTGCCAGCAGCCAAGA gattaACAGGGGagtagg GGTATGGGGGCTCCATTTCTGACTGGAGGGGTTGTG CCTGTGT

TRBV5 TGTGCCAGCAGCCAAGA tCAGGGaggg GGTATGGGGGCTCCATTTCTGACTGGAGGGGTTGTG CCTGTGT

TRBV5 TGTGCCAGCAGCCAAGA GGGGGGGCct **T**ACAGGTATGGGGGCTCCATTTCTGACTGGAGGGGTTGTG CCTGTGT

TRBV5 TGTGCCAGCAGCCAAGA**T** **C**GG GGTATGGGGGCTCCATTTCTGACTGGAGGGGTTGTG CCTGTGT

TRBV5 TGTGCCAGCAGCC GGACgggggggatt GTATGGGGGCTCCATTTCTGACTGGAGGGGTTGTG CCTGTGT

TRBV5 TGTGCCAGCAGCC gccACAGGG **TGT**ACAGGTATGGGGGCTCCATTTCTGACTGGAGGGGTTGTG CCTGTGT

TRBV5 TGTGCCAGCAGCCAAGA**T** tGGGACAG AGGTATGGGGGCTCCATTTCTGACTGGAGGGGTTGTG CCTGTGT

TRBV5 TGTGCCAGCAGCCAAGA ccGGACAGGGa ACAGGTATGGGGGCTCCATTTCTGACTGGAGGGGTTGTG CCTGTGT

TRBV5 TGTGCCAGCAGCCAAGA gAGGGGGCagg ATGGGGGCTCCATTTCTGACTGGAGGGGTTGTG CCTGTGT

TRBV5 TGTGCCAGCAGCCAAGA**T** gCAGGGG ACAGGTATGGGGGCTCCATTTCTGACTGGAGGGGTTGTG CCTGTGT

TRBV5 TGTGCCAGCAGCCAAGA agcACAG ACAGGTATGGGGGCTCCATTTCTGACTGGAGGGGTTGTG CCTGTGT

TRBV12-1 TGTGCCA ctACAGGGtaa AGGTATGGGGGCTCCATTTCTGACTGGAGGGGTTGTG CCTGTGT

TRBV12-1 TGT ccaacGACAGGGatc **GT**ACAGGTATGGGGGCTCCATTTCTGACTGGAGGGGTTGTG CCTGTGT

TRBV12-1 TGTGCCAGCTCTC cGGGACAGcttt CAGGTATGGGGGCTCCATTTCTGACTGGAGGGGTTGTG CCTGTGT

TRBV12-1 TGTGCCAGCTCTC caaGACAGGGttag GGTATGGGGGCTCCATTTCTGACTGGAGGGGTTGTG CCTGTGT

TRBV12-1 TGTGCCAGCTC cct**CC**GGGACAA ACAGGTATGGGGGCTCCATTTCTGACTGGAGGGGTTGTG CCTGTGT

TRBV12-1 TGTGCCAGCTCTCTC gaaccGGGGG ACAGGTATGGGGGCTCCATTTCTGACTGGAGGGGTTGTG CCTGTGT

TRBV12-1 TGTGCCAGCTCTCTC**CC** GGt TATGGGGGCTCCATTTCTGACTGGAGGGGTTGTG CCTGTGT

TRBV12-1 TGTGCCAGCTCCCTC **TGT** AGGTATGGGGGCTCCATTTCTGACTGGAGGGGTTGTG CCTGTGT

TRBV12-1 TGTGCCAGCTCTCT aACAGGGGGCttgg AGGTATGGGGGCTCCATTTCTGACTGGAGGGGTTGTG CCTGTGT

TRBV12-1 TGTGCCAGCTCTCTC GGGACAC ACAGGTATGGGGGCTCCATTTCTGACTGGAGGGGTTGTG CCTGTGT

TRBV12-1 TGTGCCAGCTCTCTC taAGGGtg GGGGGCTCCATTTCTGACTGGAGGGGTTGTG CCTGTGT

TRBV12-2 TGTGCCAGCTCTCTC CAGctcc ACAGGTATGGGGGCTCCATTTCTGACTGGAGGGGTTGTG CCTGTGT

TRBV12-2 TGTGCCAGCTCTCTC CAGctcc ACAGGTATGGGGCTCCATTTCTGACTGGAGGGGTTGTG CCTGTGT

TRBV12-2 TGTGCCAGCTC GCg ACAGGTATGGGGGCTCCATTTCTGACTGGAGGGGTTGTG CCTGTGT

TRBV13-1 TGTGCCAGCAGTGAT aAGGGGGG GGTATGGGGGCTCCATTTCTGACTGGAGGGGTTGTG CCTGTGT

TRBV13-1 TGTGCCAGCAGTGAT aAGGGGG GGTATGGGGGCTCCATTTCTGACTGGAGGGGTTGTG CCTGTGT

TRBV13-3 TGTGCCAGCAGTGATG tGGACcc GGTATGGGGGCTCCATTTCTGACTGGAGGGGTTGTG CCTGTGT

TRBV13-1 TGTGCCAGCAG aagGGGACA **T**ACAGGTATGGGGGCTCCATTTCTGACTGGAGGGGTTGTG CCTGTGT

TRBV13-1 TGTGCCAGCAGTGATG caaatACAGtacaa ACAGGTATGGGGGCTCCATTTCTGACTGGAGGGGTTGTG CCTGTGT

TRBV13-1 TGTGCCAGCAGTGAT ttGGGACAGGGG AGGTATGGGGGCTCCATTTCTGACTGGAGGGGTTGTG CCTGTGT

TRBV13-1 TGTGCCAGCAGTGATG ttACAGGag GGTATGGGGGCTCCATTTCTGACTGGAGGGGTTGTG CCTGTGT

TRBV13-1 TGTGCCAGCAG ccccc**CC**GGGACAGGGGt TATGGGGGCTCCATTTCTGACTGGAGGGGTTGTG CCTGTGT

TRBV13-1 TGTGCCAGCAGTGA ctctGACAGGGGGC GGTATGGGGGCTCCATTTCTGACTGGAGGGGTTGTG CCTGTGT

TRBV13-1 TGTGCCAGCAGGGA CAGGt AGGTATGGGGGCTCCATTTCTGACTGGAGGGGTTGTG CCTGTGT

TRBV13-1 TGTGCCAGCAGTGA **CC**GGGACAGGG GGTATGGGGGCTCCATTTCTGACTGGAGGGGTTGTG CCTGTGT

TRBV13-1 TGTGCCAGCAGTGATG caaaGGGACAGGG **T**ACAGGTATGGGGGCTCCATTTCTGACTGGAGGGGTTGTG CCTGTGT

TRBV13-1 TGTGCCAGCAGTGA AGGttcat **T**ACAGGTATGGGGGCTCCATTTCTGACTGGAGGGGTTGTG CCTGTGT

TRBV13-2 TGTGCCAGCGG **CC**GGGACAGGGt **T**ACAGGTATGGGGGCTCCATTTCTGACTGGAGGGGTTGTG CCTGTGT

TRBV13-2 TGTGCCAGCGGTG ct ACAGGTATGGGGGCTCCATTTCTGACTGGAGGGGTTGTG CCTGTGT

TRBV13-2 TGTGCCAGCGG aaACAGGGGc GTATGGGGGCTCCATTTCTGACTGGAGGGGTTGTG CCTGTGT

TRBV13-2 TGTGCCAACGG aaACAGGGGc GTATGGGGGCTCCATTTCTGACTGGAGGGGTTGTG CCTGTGT

TRBV13-2 TGTGCCAGCGG gGGGACAGGGGG GGTATGGGGGCTCCATTTCTGACTGGAGGGGTTGTG CCTGTGT

TRBV13-2 TGTGCCAGCGG GGG GGTATGGGGGCTCCATTTCTGACTGGAGGGGTTGTG CCTGTGT

TRBV13-2 TGTGCCAGCGG GG GGTATGGGGGCTCCATTTCTGACTGGAGGGGTTGTG CCTGTGT

TRBV13-2 TGTGCCAGCGGTGA GAAC ACAGGTATGGGGGCTCCATTTCTGACTGGAGGGGTTGTG CCTGTGT

TRBV13-2 TGTGCCAGCGGTGAT tGACAGGGGGCtcg GGTATGGGGGCTCCATTTCTGACTGGAGGGGTTGTG CCTGTGT

TRBV13-2 TGTGCCAGCGGTGAT taACAGGGGGCtcg GGTATGGGGGCTCCATTTCTGACTGGAGGGGTTGTG CCTGTGT

TRBV13-2 TGTGCCAGCGG attACAGGGGGCG GGTATGGGGGCTCCATTTCTGACTGGAGGGGTTGTG CCTGTGT

TRBV13-2 TGTGCCAGCGGTGATG **CA**GGGACAGGGc **T**ACAGGTATGGGGGCTCCATTTCTGACTGGAGGGGTTGTG CCTGTGT

TRBV13-2 TGTGCCAGCGGTGATG CtGGGAGT ACAGGTATGGGGGCTCCATTTCTGACTGGAGGGGTTGTG CCTGTGT

TRBV13-3 TGTGCCAGCAGTG **CC**GGGACAGGGGaga GTATGGGGGCTCCATTTCTGACTGGAGGGGTTGTG CCTGTGT

TRBV13-3 TGTGCCAGCAGTGA CAGGG AGGTATGGGGGCTCCATTTCTGACTGGAGGGGTTGTG CCTGTGT

TRBV13-3 TGTGCCAGCAGTGA cGGG **T**ACAGGTATGGGGGCTCCATTTCTGACTGGAGGGGTTGTG CCTGTGT

TRBV13-3 TGTGCCAGCAGTGA AGGGGGgcag GGTATGGGGGCTCCATTTCTGACTGGAGGGGTTGTG CCTGTGT

TRBV13-3 TGTGCCAGCAG GGt CAGGTATGGGGGCTCCATTTCTGACTGGAGGGGTTGTG CCTGTGT

TRBV13-3 TGTGCCAGCAGTGATG cACAGagg AGGTATGGGGGCTCCATTTCTGACTGGAGGGGTTGTG CCTGTGT

TRBV13-3 TGTGCCAGCAGT CAGGGttggg GGTATGGGGGCTCCATTTCTGACTGGAGGGGTTGTG CCTGTGT

TRBV13-3 TGTGCCAGCAGTG gtccccGC GGGGCTCCATTTCTGACTGGAGGGGTTGTG CCTGTGT

TRBV13-3 TGTGCCAGCAGTGA GACAGGGc GGGGGCTCCATTTCTGACTGGAGGGGTTGTG CCTGTGT

TRBV14 TGTGCCCCGG GGTATGGGGGCTCCATTTCTGACTGGAGGGGTTGTG CCTGTGT

TRBV14 TGTGCCAGCAGT cgggCAGGca **GT**ACAGGTATGGGGGCTCCATTTCTGACTGGAGGGGTTGTG CCTGTGT

TRBV14 TGTGCCAGCAGTTTC aGGGA **GT**ACAGGTATGGGGGCTCCATTTCTGACTGGAGGGGTTGTG CCTGTGT

TRBV14 TGTGCCAGCAG ccgactcaAGGG **T**ACAGGTATGGGGGCTCCATTTCTGACTGGAGGGGTTGTG CCTGTGT

TRBV15 TGTGCCAGCAGTTTAG tCAGGcctc GGGGGCTCCATTTCTGACTGGAGGGGTTGTG CCTGTGT

TRBV15 TGTGCCAGCAGT cACAGGGGtgg GGTATGGGGGCTCCATTTCTGACTGGAGGGGTTGTG CCTGTGT

TRBV15 TGTGCCAGCAGTTTAG GGACAGGGG GGTATGGGGGCTCCATTTCTGACTGGAGGGGTTGTG CCTGTGT

TRBV15 TGTGCCAGCAG aatcACAGGGG ACAGGTATGGGGGCTCCATTTCTGACTGGAGGGGTTGTG CCTGTGT

TRBV15 TGTGCCAGCAG ccaAGG GGTATGGGGGCTCCATTTCTGACTGGAGGGGTTGTG CCTGTGT

TRBV15 TGTGCCAGCAG ggcCAGGG **T**ACAGGTATGGGGGCTCCATTTCTGACTGGAGGGGTTGTG CCTGTGT

TRBV15 TGTGCCAGCAG cccccGACcg CAGGTATGGGGGCTCCATTTCTGACTGGAGGGGTTGTG CCTGTGT

TRBV15 TGTGCCAGCAGTTTAGC**G** **CC**GGGAat GGTATGGGGGCTCCATTTCTGACTGGAGGGGTTGTG CCTGTGT

TRBV15 TGTGCCAGCAGTTTAGC**G** **CC**GGGAat GGTATGGGGGCTCCATTTCTGACTGGAGGGGTTGTG CCTGTGT

TRBV15 TGTGCCAGCAGT ccatcaa **GT**ACAGGTATGGGGGCTCCATTTCTGACTGGAGGGGTTGTG CCTGTGT

TRBV16 TGTGCAAGCAGCTTAGA**TC** aGGGACAGGGagt AGGTATGGGGGCTCCATTTCTGACTGGAGGGGTTGTG CCTGTGT

TRBV16 TGTGCAAGCAGTT caGGACAGGG GGTATGGGGGCTCCATTTCTGACTGGAGGGGTTGTG CCTGTGT

TRBV16 TGTGCAAGCAGCTTAGA CAGaa GTATGGGGGCTCCATTTCTGACTGGAGGGGTTGTG CCTGTGT

TRBV16 TGTGCAAGCAGCTTAGA**T** gaGACAa ACAGGTATGGGGGCTCCATTTCTGACTGGAGGGGTTGTG CCTGTGT

TRBV16 TGTGCAAGCAGCTTAGA atcGGGAg GGTATGGGGGCTCCATTTCTGACTGGAGGGGTTGTG CCTGTGT

TRBV16 TGTGCAAGCAGCTTAG GGGGGa GGTATGGGGGCTCCATTTCTGACTGGAGGGGTTGTG CCTGTGT

TRBV17 TGTGCTAGCAGT GGACT **T**ACAGGTATGGGGGCTCCATTTCTGACTGGAGGGGTTGTG CCTGTGT

TRBV17 TGTGCTAGCAGTAGA ggCAGGGGagag GGTATGGGGGCTCCATTTCTGACTGGAGGGGTTGTG CCTGTGT

TRBV17 TGTGCTAGCAGTAG tGGGACAGGGGc ACAGGTATGGGGGCTCCATTTCTGACTGGAGGGGTTGTG CCTGTGT

TRBV17 TGTGCTAGCAGTAGAGA CAGGGtcctc ACAGGTATGGGGGCTCCATTTCTGACTGGAGGGGTTGTG CCTGTGT

TRBV17 TGTGCTAGCAGTAG tACAGGGtt GGTATGGGGGCTCCATTTCTGACTGGAGGGGTTGTG CCTGTGT

TRBV19 TGTGCCAGCAGTA aggCAGtt GGTATGGGGGCTCCATTTCTGACTGGAGGGGTTGTG CCTGTGT

TRBV19 TGTGCCAGCAGTAT gAGGGGGC**G** GGTATGGGGGCTCCATTTCTGACTGGAGGGGTTGTG CCTGTGT

TRBV19 TGTGCCAGCAGTAT cCAGGGGGC**GC**tggg GGGGGCTCCATTTCTGACTGGAGGGGTTGTG CCTGTGT

TRBV19 TGTGCCAGCAGTAT cCAGGGGGC**GC**tgggg GGGGGCTCCATTTCTGACTGGAGGGGTTGTG CCTGTGT

TRBV19 TGTGCCAGCA tGACAGGGGGCt GTATGGGGGCTCCATTTCTGACTGGAGGGGTTGTG CCTGTGT

TRBV19 TGTGCCAGCAGTATA CAGGGGGC**GC**ag GGTATGGGGGCTCCATTTCTGACTGGAGGGGTTGTG CCTGTGT

TRBV19 TGTGCCAGCAGTATA aACAGGGGGat GGTATGGGGGCTCCATTTCTGACTGGAGGGGTTGTG CCTGTGT

TRBV19 TGTGCCAGCAGTAT TGGGGGGGa ACAGGTATGGGGGCTCCATTTCTGACTGGAGGGGTTGTG CCTGTGT

TRBV19 TGTGCCAGCA cCAG ACAGGTATGGGGGCTCCATTTCTGACTGGAGGGGTTGTG CCTGTGT

TRBV19 TGTGCCAGCAG ccGGACAGGGtcc ACAGGTATGGGGGCTCCATTTCTGACTGGAGGGGTTGTG CCTGTGT

TRBV19 TGTGCCAGC TATGGGGGCTCCATTTCTGACTGGAGGGGTTGTG CCTGTGT

TRBV19 TGTGCCAGCAGTAT cGGGGGGGa **GT**ACAGGTATGGGGGCTCCATTTCTGACTGGAGGGGTTGTG CCTGTGT

TRBV19 TGTGCCAGCAGTATAG AGccaac **TGT**ACAGGTATGGGGGCTCCATTTCTGACTGGAGGGGTTGTG CCTGTGT

TRBV19 TGTGCCAGCAGTATAG AGccaac **TGT**ACAGGTATGGGGGCTCCACTTCTGACTGGAGGGGTTGTG CCTGTGT

TRBV19 TGTGCCAGCAGT cctGGACAa ACAGGTATGGGGGCTCCATTTCTGACTGGAGGGGTTGTG CCTGTGT

TRBV19 TGTGCCAGCAGTATAG TGGGtgg GGGGGCTCCATTTCTGACTGGAGGGGTTGTG CCTGTGT

TRBV20 TGTGGTGCT gCGGGACAaa **T**ACAGGTATGGGGGCTCCATTTCTGACTGGAGGGGTTGTG CCTGTGT

TRBV20 TGTGGTGCTAGGGA ggcCAGGGag AGGTATGGGGGCTCCATTTCTGACTGGAGGGGTTGTG CCTGTGT

TRBV20 TGTGGTGCTAGGGA**TC** tcGACAGGact GGTATGGGGGCTCCATTTCTGACTGGAGGGGTTGTG CCTGTGT

TRBV20 TGTGGTG tccGGGGACAGaa ATGGGGGCTCCATTTCTGACTGGAGGGGTTGTG CCTGTGT

TRBV20 TGTGGTGCT tagacgCAGGGGGa ACAGGTATGGGGGCTCCATTTCTGACTGGAGGGGTTGTG CCTGTGT

TRBV20 TGTGGTGCTA tccGGGACA AGGTATGGGGGCTCCATTTCTGACTGGAGGGGTTGTG CCTGTGT

TRBV20 TGTGGTGCTAGGGA**TC** aAGGGGGgcgaggagga TATGGGGGCTCCATTTCTGACTGGAGGGGTTGTG CCTGTGT

TRBV20 TGTGGTGC agCAGGGG ACAGGTATGGGGGCTCCATTTCTGACTGGAGGGGTTGTG CCTGTGT

TRBV20 TGTGGTGCTA AG ACAGGTATGGGGGCTCCATTTCTGACTGGAGGGGTTGTG CCTGTGT

TRBV20 TGTGGTGCTAG **CC**GGG AGGTATGGGGGCTCCATTTCTGACTGGAGGGGTTGTG CCTGTGT

TRBV21 TGTGCTAGCAGTCAATC **G**tACAGGGc GGTATGGGGGCTCCATTTCTGACTGGAGGGGTTGTG CCTGTGT

TRBV21 TGTGCTAGCAGTCAATC gt **TGT**ACAGGTATGGGGGCTCCATTTCTGACTGGAGGGGTTGTG CCTGTGT

TRBV24 TGTGCCAGCAGTCTG GGGACAGGGa ACAGGTATGGGGGCTCCATTTCTGACTGGAGGGGTTGTG CCTGTGT

TRBV24 TGTGCCAGCAGTCT tttGGACAtaa TATGGGGGCTCCATTTCTGACTGGAGGGGTTGTG CCTGTGT

TRBV24 TGTGCCAGCAGTC ctaGGGACAGaaggg GGTATGGGGGCTCCATTTCTGACTGGAGGGGTTGTG CCTGTGT

TRBV24 TGTGCCAGCAGCC ccGGACAGGGag GGTATGGGGGCTCCATTTCTGACTGGAGGGGTTGTG CCTGTGT

TRBV24 TGTGCCAGCAGTCTGT GGGAtg GGTATGGGGGCTCCATTTCTGACTGGAGGGGTTGTG CCTGTGT

TRBV24 TGTGCCAGCAGT tctACAGGGGagag GGTATGGGGGCTCCATTTCTGACTGGAGGGGTTGTG CCTGTGT

TRBV24 TGTGCCAGCAGTCTGT GACAa ACAGGTATGGGGGCTCCATTTCTGACTGGAGGGGTTGTG CCTGTGT

TRBV24 TGTGCCAGCAG GGACAaa ACAGGTATGGGGGCTCCATTTCTGACTGGAGGGGTTGTG CCTGTGT

TRBV24 TGTGCCAGCAGTCTGT tccCAGGtg GGTATGGGGGCTCCATTTCTGACTGGAGGGGTTGTG CCTGTGT

TRBV24 TGTGCCAGCAGTCT taccACAGGGGG **GT**ACAGGTATGGGGGCTCCATTTCTGACTGGAGGGGTTGTG CCTGTGT

TRBV24 TGTGCCAGCAGTCT **CC**GGACAGGGGaa TATGGGGGCTCCATTTCTGACTGGAGGGGTTGTG CCTGTGT

TRBV24 TGTGCCAGCAGTCTGT GACAGGGctct **T**ACAGGTATGGGGGCTCCATTTCTGACTGGAGGGGTTGTG CCTGTGT

TRBV26 TGTGCCAGCAG ccctGACAGGa ACAGGTATGGGGGCTCCATTTCTGACTGGAGGGGTTGTG CCTGTGT

TRBV26 TGTGCCAGCAGTC **C**GGGACAatc **GT**ACAGGTATGGGGGCTCCATTTCTGACTGGAGGGGTTGTG CCTGTGT

TRBV26 TGTGCCAGCAGTCTG GACAc ACAGGTATGGGGGCTCCATTTCTGACTGGAGGGGTTGTG CCTGTGT

TRBV26 TGTGCCAGCAGTCTG GGGGGG AGGTATGGGGGCTCCATTTCTGACTGGAGGGGTTGTG CCTGTGT

TRBV26 TGTGCCAGCAGTCTGTC CAG **T**ACAGGTATGGGGGCTCCATTTCTGACTGGAGGGGTTGTG CCTGTGT

TRBV26 TGTGCCAGCAGTC **CCC**GGGAC ACAGGTATGGGGGCTCCATTTCTGACTGGAGGGGTTGTG CCTGTGT

TRBV26 TGTGCCAGCAGTCTGT GGGGGGGCaa GTATGGGGGCTCCATTTCTGACTGGAGGGGTTGTG CCTGTGT

TRBV26 TGTGCCAGCAGTC gcGACAGt **T**ACAGGTATGGGGGCTCCATTTCTGACTGGAGGGGTTGTG CCTGTGT

TRBV29 TGTGCTAGCAGTTTATC**G** gtGG GGTATGGGGGCTCCATTTCTGACTGGAGGGGTTGTG CCTGTGT

TRBV29 TGTGCTAGCAGTT cttacGGACAGGGc GGGTATGGGGGCTCCATTTCTGACTGGAGGGGTTGTG CCTGTGT

TRBV29 TGTGCTAGCAG a**CC**GGGACAG CAGGTATGGGGGCTCCATTTCTGACTGGAGGGGTTGTG CCTGTGT

TRBV29 TGTGCTAGCAG GGACAGGGG AGGTATGGGGGCTCCATTTCTGACTGGAGGGGTTGTG CCTGTGT

TRBV29 TGTGCTAGCAGTTTATC GGA AGGTATGGGGGCTCCATTTCTGACTGGAGGGGTTGTG CCTGTGT

TRBV29 TGTGCTAGCAGTTT ttttt GGTATGGGGGCTCCATTTCTGACTGGAGGGGTTGTG CCTGTGT

TRBV29 TGTGCTAGCAGTTT gGGGACAGGGag GGGGGCTCCATTTCTGACTGGAGGGGTTGTG CCTGTGT

TRBV29 TGTGCTAGC GACAGGGGGtag GGTATGGGGGCTCCATTTCTGACTGGAGGGGTTGTG CCTGTGT

TRBV30 TGTAGTTCTA CAGG GGTATGGGGGCTCCATTTCTGACTGGAGGGGTTGTG CCTGTGT

TRBV30 TGTAGTTCTAGA **CC**GGGACAGGGGG CAGGTATGGGGGCTCCATTTCTGACTGGAGGGGTTGTG CCTGTGT

TRBV30 TGTAGTTCTAG cgGGGAAGGGGagag AGGTATGGGGGCTCCATTTCTGACTGGAGGGGTTGTG CCTGTGT

TRBV31 TGTGCCTGGAGTCT agaGGGACAGGGG ACAGGTATGGGGGCTCCATTTCTGACTGGAGGGGTTGTG CCTGTGT

TRBV31 TGTGCCTGGA cccctaaGACAGGGtcg GGTATGGGGGCTCCATTTCTGACTGGAGGGGTTGTG CCTGTGT

TRBV31 TGTGCCTGGAG aGGGACAGGGacgg AGGTATGGGGGCTCCATTTCTGACTGGAGGGGTTGTG CCTGTGT

TRBV31 TGTGCCTGGAGTC GGGG **GT**ACAGGTATGGGGGCTCCATTTCTGACTGGAGGGGTTGTG CCTGTGT

TRBV31 TGTGCCTGGAGT gtgaACAGGGGGCgcat GTATGGGGGCTCCATTTCTGACTGGAGGGGTTGTG CCTGTGT

TRBV31 TGTGCCTGGAGT gtgaACAGGGGGCgcgt GTATGGGGGCTCCATTTCTGACTGGAGGGGTTGTG CCTGTGT

TRBV31 TGTGCCTGGAG gcgcGACAagagg GGTATGGGGGCTCCATTTCTGACTGGAGGGGTTGTG CCTGTGT

**Sequences with retention of GL DNA beyond the -40 RSS**

TRBV3 TGTGCCAGCAGCT (CAGGCACCCGGCTCACTGTG)ACAGGTATGGGGGCTCCATTTCTGACTGGAGGGGTTGTGCCTGTGT

TRBV1 TGCACCTGCAGGACAG at (ACCCGGCTCACTGTG)ACAGGTATGGGGGCTCCATTTCTGACTGGAGGGGTTGTGCCTGTGT

TRBV3 TGTGCCAGCAGCTT GACAGG (AGGCACCCGGCTCACTGTG)ACAGGTATGGGGGCTCCATTTCTGACTGGAGGGGTTGTGCCTGTGT

TRBV12 TGTGCCAGCTCT accGGAa (CGGCTCACTGTG)ACAGGTATGGGGGCTCCATTTCTGACTGGAGGGGTTGTGCCTGTGT
